# White Blood Cells and Blood Pressure: A Mendelian Randomization Study

**DOI:** 10.1161/CIRCULATIONAHA.119.045102

**Published:** 2020-03-09

**Authors:** Mateusz Siedlinski, Ewelina Jozefczuk, Xiaoguang Xu, Alexander Teumer, Evangelos Evangelou, Renate B. Schnabel, Paul Welsh, Pasquale Maffia, Jeanette Erdmann, Maciej Tomaszewski, Mark J. Caulfield, Naveed Sattar, Michael V. Holmes, Tomasz J. Guzik

**Affiliations:** 1 Department of Internal and Agricultural Medicine, Faculty of Medicine, Jagiellonian University Medical College, Krakow, Poland (M.S., E.J., T.J.G.).; 2 Institute of Cardiovascular and Medical Sciences (M.S., P.W., N.S., T.J.G.), University of Glasgow, United Kingdom.; 3 Institute of Infection, Immunity, and Inflammation (P.M.), University of Glasgow, United Kingdom.; 4 Division of Cardiovascular Sciences, School of Medical Sciences, Faculty of Biology, Medicine and Health, University of Manchester, United Kingdom (X.X., M.T.).; 5 Department SHIP/Clinical-Epidemiological Research, Institute for Community Medicine, University Medicine Greifswald, Germany (A.T.).; 6 German Centre for Cardiovascular Research partner site Greifswald, Germany (A.T.).; 7 Department of Epidemiology and Biostatistics, School of Public Health, Imperial College London, United Kingdom (E.E.).; 8 University Heart Center Hamburg Eppendorf, German Center for Cardiovascular Research partner site Hamburg/Kiel/Lübeck, Germany (R.B.S.).; 9 Department of Pharmacy, University of Naples Federico II, Italy (P.M.).; 10 Institute for Cardiogenetics, University of Lübeck, Germany (J.E.).; 11 William Harvey Research Institute, National Institute for Health Research Biomedical Research Centre at Barts, Queen Mary University of London, United Kingdom (M.J.C.).; 12 Medical Research Council Population Health Research Unit, Clinical Trial Service Unit and Epidemiological Studies Unit, Nuffield Department of Population Health, University of Oxford, United Kingdom (M.V.H.).

**Keywords:** blood pressure, hypertension, Mendelian randomization analysis, white blood cells

## Abstract

**Background:**

High blood pressure (BP) is a risk factor for cardiovascular morbidity and mortality. While BP is regulated by the function of kidney, vasculature, and sympathetic nervous system, recent experimental data suggest that immune cells may play a role in hypertension.

**Methods:**

We studied the relationship between major white blood cell types and blood pressure in the UK Biobank population and used Mendelian randomization (MR) analyses using the ≈750 000 UK-Biobank/International Consortium of Blood Pressure-Genome-Wide Association Studies to examine which leukocyte populations may be causally linked to BP.

**Results:**

A positive association between quintiles of lymphocyte, monocyte, and neutrophil counts, and increased systolic BP, diastolic BP, and pulse pressure was observed (eg, adjusted systolic BP mean±SE for 1st versus 5th quintile respectively: 140.13±0.08 versus 141.62±0.07 mm Hg for lymphocyte, 139.51±0.08 versus 141.84±0.07 mm Hg for monocyte, and 137.96±0.08 versus 142.71±0.07 mm Hg for neutrophil counts; all *P*<10^–50^). Using 121 single nucleotide polymorphisms in MR, implemented through the inverse-variance weighted approach, we identified a potential causal relationship of lymphocyte count with systolic BP and diastolic BP (causal estimates: 0.69 [95% CI, 0.19–1.20] and 0.56 [95% CI, 0.23–0.90] of mm Hg per 1 SD genetically elevated lymphocyte count, respectively), which was directionally concordant to the observational findings. These inverse-variance weighted estimates were consistent with other robust MR methods. The exclusion of rs3184504 SNP in the *SH2B3* locus attenuated the magnitude of the signal in some of the MR analyses. MR in the reverse direction found evidence of positive effects of BP indices on counts of monocytes, neutrophils, and eosinophils but not lymphocytes or basophils. Subsequent MR testing of lymphocyte count in the context of genetic correlation with renal function or resting and postexercise heart rate demonstrated a positive association of lymphocyte count with urine albumin-to-creatinine ratio.

**Conclusions:**

Observational and genetic analyses demonstrate a concordant, positive and potentially causal relationship of lymphocyte count with systolic BP and diastolic BP.

## Abstract 2

Supplemental Digital Content is available in the text.

**Clinical Perspective**

**What Is New?**

- This cross-sectional and genetic study found potentially causal, positive effects of total blood lymphocyte count with regards to blood pressure.
- Among mechanisms that might mediate this relationship, we found evidence that blood lymphocyte count might influence albuminuria.
- This study may also support a reverse, potentially causal positive effect of blood pressure indices on blood neutrophil, monocyte, and eosinophil counts.

**What Are the Clinical Implications?**

- High blood lymphocyte count may play a causal role in the development of hypertension.

High blood pressure (BP) causes cardiovascular disease, including coronary heart disease (CHD) and stroke. While classical BP regulation is controlled by the function of kidney, vasculature and sympathetic nervous system, recent experimental data suggest that immune cells may play an important role in the development of hypertension.^1–5^ For example, immune cells infiltrating heart,^6^ perivascular adipose tissue, or kidneys contribute to dysfunction of these organs and can mediate high BP.^2^ *RAG1*^–/–^ mice^7^ or rats,^8^ lacking functional T and B lymphocytes, are protected from experimental hypertension, and transfer of T lymphocytes restores the hypertensive phenotype.^7^ Moreover, targeting B cells,^9^ monocytes, and neutrophils as well as targeting inflammasome^10–13^ shows their contribution to vessel remodeling and hypertension. While most data originate from experimental models, the relationships between white blood cells and hypertension remains poorly defined in humans.

Well-powered, genome-wide association studies (GWAS) have identified hundreds of single nucleotide polymorphisms (SNPs) associated with BP-related traits or circulating blood cell indices.^14,15^ This creates an opportunity to test genetic, potentially causal relationships between these and other, clinically relevant cardiovascular traits using the Mendelian randomization (MR) approach. In brief, MR exploits characteristics of the human genome—principally its random allocation and nonmodifiable nature—to make potentially causal deductions on the relationship of an exposure on a trait or disease.^16^ Indeed, previous studies using such genetic approaches identified evidence in support of a causal relationship between white blood cell parameters such as lymphocytes and risk of CHD, making the exploration of the role of white blood cells in altering BP of particular interest.^15^

In the present study, we studied the relationship between major white blood cell types and BP in the UK Biobank (UKB) population and used MR analysis using data generated from large GWAS to examine whether leukocyte subpopulations may be causally linked to BP.

## Methods

UKB data are available on application for data access (http://www.ukbiobank.ac.uk/). Data concerning SNPs used in MR analyses, linking white blood cell count and BP indices, are available in the Excel file in the Data Supplement. GWAS summary statistics of the CKDGen (Chronic Kidney Disease Genetics) Consortium (http://ckdgen.imbi.uni-freiburg.de/), white blood cell counts (http://www.bloodcellgenetics.org/), heart rate (HR) phenotypes (https://www.cardiomics.net/download-data, https://data.mendeley.com/datasets/tg5tvgm436/1) and BP indices (UKB+ICBP [International Consortium for Blood Pressure]; https://www.ebi.ac.uk/gwas/downloads/summary-statistics) are available online. The ICBP GWAS summary statistics can be assessed through the ICBP Consortium.

### Testing the Observational Association Between Blood Cell Counts and BP Indices

UKB recruited 502 639 participants (37 to 73 years of age) from 22 assessment centers across the United Kingdom between 2007 and 2010. Baseline biological measurements were recorded, and touchscreen questionnaires were administered, as described elsewhere.^17^ UKB received ethical approval from the North West Multi-Center Research Ethics Committee (11/NW/03820). All participants gave written informed consent before enrollment in the study, which was conducted in accordance with the principles of the Declaration of Helsinki. Using self-reported, ethnic background information, we selected 442 604 white British participants of the UK Biobank study to assess the effect of quintiles of circulating monocyte, lymphocyte, eosinophil, neutrophil, and basophil counts on systolic BP (SBP), diastolic BP (DBP), and pulse pressure (PP).^17^ Whereas blood cell indices often correlate with each other (Table I in the Data Supplement), these 5 subpopulations were among 13, out of 36, blood cell indices selected by Astle et al as relatively independent and approximated all other white blood cell indices including absolute and percentage counts (eg, blood neutrophil count strongly correlated with total white blood cell count and percentage of lymphocytes).^15,18^

The average of 2 automated, sitting BP readings (see Data Supplement for details regarding BP measurement procedure) was calculated and used in final analyses, and individuals with only 1 reading available (9.2% for SBP or DBP) were excluded from further analyses, leaving 384 721 individuals with available white blood cell counts for final analysis (Table). Repeated blood pressure measurements were highly correlated.^19^

**Table.**
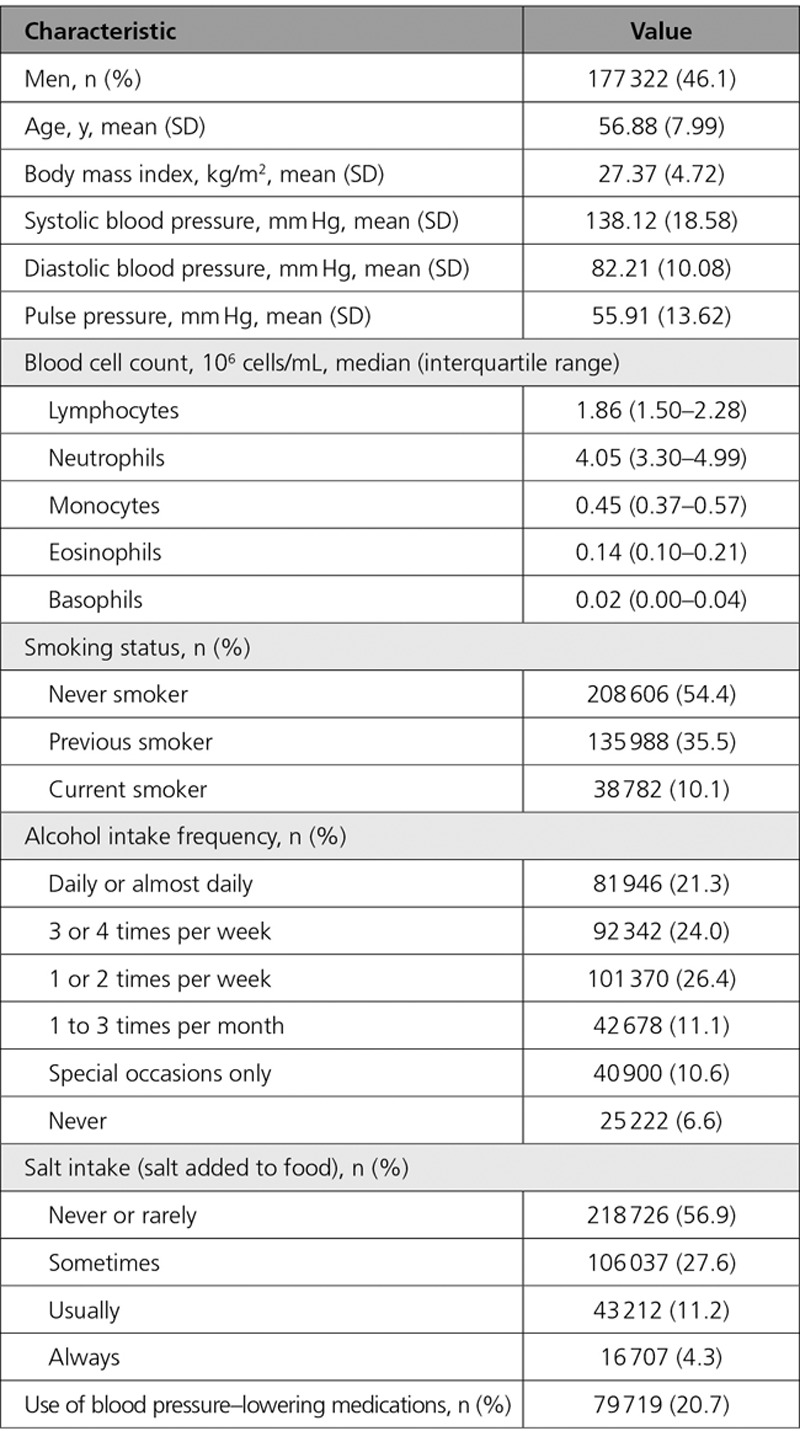
Characteristics of 384 721 White, British Participants From the UK Biobank Study Who Were Included in the Analysis

BP values from individuals on BP-lowering medications were adjusted in the same way as compared with GWAS on BP indices in the UKB and ICBP^14,20^ (ie, by adding 15 and 10 mm Hg to SBP and DBP, respectively;^21^ see Figure I in the Data Supplement for distribution of BP indices). We also performed separate sensitivity analyses after exclusion of individuals reporting BP-lowering medication use, using the same quintile boundaries as compared with the whole cohort. White blood cells were measured on fresh samples as an absolute number per unit volume, and their component leukocytes (lymphocytes, monocytes, neutrophils, eosinophils, and basophils) as absolute measures and proportions of the overall white blood cells; all using an automated, clinically validated, Coulter LH 750. Calibration and quality control were performed in line with the manufacturer’s recommendations.^18^ Further details of these measurements can be found in the UK Biobank online showcase and protocol (http://www.ukbiobank.ac.uk). While quintiles of monocyte, lymphocyte, eosinophil, and neutrophil counts contained, on average, 76 944±4588 subjects, quintiles of basophil count were less balanced, with 96 897 subjects (all subjects with no basophils detected), 31 792, 128 937, 55 910, and 77 185 individuals in the 1st, 2nd, 3rd, 4th, and 5th quintiles, respectively (see Figure II in the Data Supplement for distributions of white blood cell counts).

### MR Analyses Testing Causal Effect of Blood Cell Count on BP Indices

#### Instrumental Variables

We selected SNPs that associated with the level of circulating monocyte, lymphocyte, eosinophil, basophil, or neutrophil counts at genome-wide significance (*P*<8.31×10^–9^) in analysis of UKB and INTERVAL studies comprising 173 480 individuals.^15^ Final analyses included common (minor allele frequency>5%) and uncorrelated (*r*^2^<0.2; validated in European British panel using LDlink)^22^ SNPs available in GWAS on outcome variables. Palindromic SNPs with a minor allele frequency>40% in the exposure GWAS, as well as SNPs from the major histocompatibility complex region (chr6:20 000 000–40 000 000, GRCh37), which likely possess pleiotropic effects, were excluded from analyses.

#### Outcome Variables

Our primary analysis examined whether there exists a relation between 5 selected blood cell indices and SBP, DBP, or PP on the basis of genetic effect estimates published by Evangelou et al in meta-analysis of 2 independent GWAS, the UKB and ICBP, comprising 757 601 individuals.^14^ We estimated that approximately 17.6% of individuals from the UKB+ICBP GWAS^14^ were also included in the GWAS on blood cell counts.^15^ Thus, to ensure that this overlap did not overly influence our results (eg, because of overfitting or winner’s curse),^16^ a sensitivity analysis was performed using GWAS estimates derived solely from the ICBP consortium comprising 299 024 individuals.^14^ Of note, GWAS analysis on BP indices was adjusted for body mass index,^14^ while GWAS analysis on blood cell counts was adjusted for log-height and log-weight, which implicitly adjusted for body mass index.^15^ Therefore both GWAS of exposures and outcomes adjusted for adiposity-related traits, potentially limiting confounding caused by collider bias.^23^

A secondary analysis aimed to identify an intermediate phenotype, which may mediate the causal effects, if present, of lymphocyte count on BP. Therefore, using published GWAS summary data, we selected traits related to kidney and heart function that have previously demonstrated a causal link to BP or hypertension through genetic analyses. This included resting HR,^24^ increase in HR from resting level to peak exercise level,^25^ estimated glomerular filtration rate assessed in individuals of European ancestry,^26^ and urine albumin-to-creatinine ratio (UACR) derived from 2 different studies, that is, the CKDGen Consortium, and meta-analysis of the UK Biobank and CKDGen consortium.^27,28^ All participants, included in the individual studies within the CKDGen and ICBP consortia, provided written informed consent and studies were approved by their local research ethics committees and institutional review boards as applicable.^20,26–28^

### Reverse MR Analyses

We considered 885 SNPs replicated in 1- or 2-stage analyses in a GWAS on BP indices by Evangelou et al.^14^ Among these, we selected 883 independent SNPs (*r*^2^<0.2 validated in the European British panel using LDlink),^22^ and associated with SBP, DBP, or PP at *P*<5×10^–8^ in the meta-analysis of the UKB and ICBP consortia,^14^ and used these as instrumental variables (IVs) in an MR analysis on 5 selected blood cell indices.^15^ Palindromic SNPs with a minor allele frequency >40% in the exposure GWAS, as well as SNPs from the major histocompatibility complex region, were excluded from these analyses.

### Statistical Analysis

General linear model in SPSS (version 25.0) was used to test the association of quintiles of cell counts on SBP, DBP, and PP level while adjusting for sex, age, age squared, body mass index, smoking status (3 categories: never, former, and current), and alcohol intake frequency (6 categories: never, special occasions only, 1 to 3 times a month, 1 or 2 times a week, 3 or 4 times a week, and daily or almost daily). Quintile-specific estimated marginal means were reported and compared using analysis of variance. To verify the observed associations, we performed similar analyses with additional adjustment for salt intake (Table 1)^1,29^ or using quantile regression using quantreg package in R (version 3.6.2) with the 3rd quintile set as a reference. To model the continuous relationship between blood cell counts and medication-adjusted BP indices quantile regression, the generalized additive model using mgcv package (version 1.8–31),^30^ and general linear model analyses in R were used. Cubic regression spline smooth was applied to cell count parameters and generalized additive model analyses were also adjusted for body mass index, age, age squared, sex, smoking status, and alcohol intake frequency.

Inverse-variance weighted (IVW) analyses were performed using MendelianRandomization package in R.^31^ Additional sensitivity analyses were performed using methods that are more robust to violations of MR assumptions, that is, weighted median approach, allowing up to 50% of the weights to be invalid IVs,^31^ MR-Egger method, which can include SNPs with pleiotropic effects that are not proportional to the effects of these SNPs on exposure,^16,31^ as well as MR-PRESSO (Mendelian Randomization Pleiotropy Residual Sum and Outlier) method, which identifies and excludes SNPs that most likely display pleiotropic effects.^32^ Leave-one-out sensitivity analyses were performed using TwoSampleMR package in R.^33^ False discovery rate–based correction was applied to *P* values derived from MR analyses.

## Results

### Levels of White Blood Cell Subpopulations Associate With BP in the UKB

**Figure 1.**
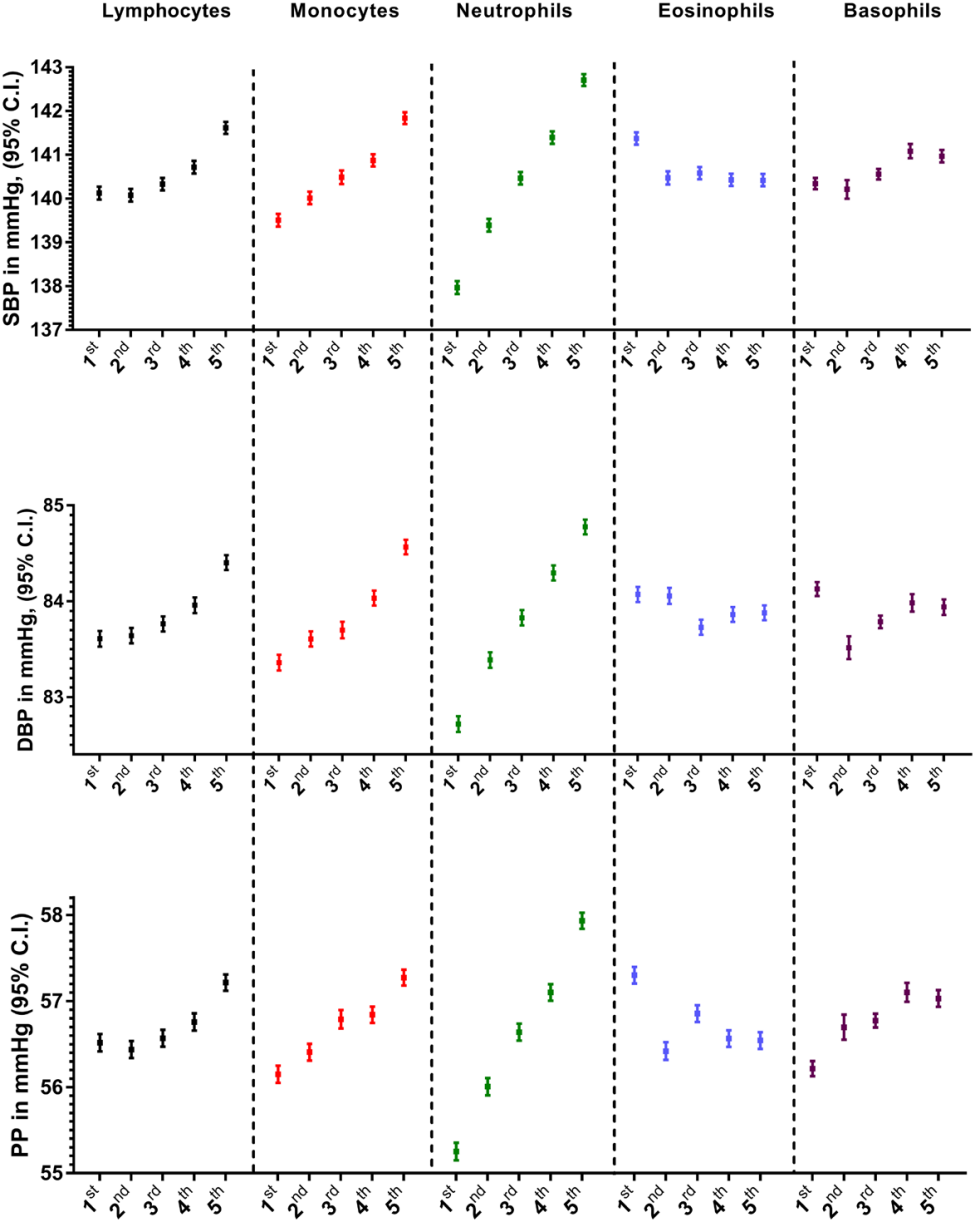
**Levels of 5 white blood cell types are associated with systolic blood pressure (SBP), diastolic blood pressure (DBP), and pulse pressure (PP) in the UK Biobank.** Estimated marginal means of blood pressure indices, from general linear model analysis adjusted for sex, age, age squared, body mass index, smoking status, and alcohol intake frequency, are presented according to quintiles of counts of white blood cell subpopulations. All ANOVA tests, assessing global between-quintile differences in blood pressure indices were significant at *P*<10^–11^. Post hoc tests revealed that all comparisons between the 1st and the 5th quintile of any cell type count with respect to any blood pressure index were significant at Bonferroni-corrected *P*<0.05, given 150 tests (5 types of blood cell counts×3 blood pressure indices×10 between-quintile differences) performed.

An association between quintiles of all cell types and SBP, DBP, and PP was observed (Figure 1). While the differences between quintiles showed strong evidence of associations, even after Bonferroni correction for multiple testing, the association of blood neutrophil count with all 3 BP indices was the strongest, relatively, as compared with the other white blood cells analyzed (eg, difference in adjusted SBP between the 5th and 1st quintile in mm Hg [95% CI]: 4.74 for neutrophil [4.47–5.01], 2.33 for monocyte, [2.06–2.60], 1.49 for lymphocyte [1.22–1.76], 0.63 for basophil [0.37–0.88], and −0.95 for eosinophil counts [−1.21 to −0.69]; Figure 1). This was further supported by analysis of continuously defined white blood cell counts (Table II in the Data Supplement).

Of note, the 1st quintile of eosinophil count was associated with higher SBP and PP compared with any other quintile (Figure 1). Associations identified in the aforementioned observational analyses using general linear model were confirmed using quantile regression analysis (Table III in the Data Supplement), after exclusion of individuals on BP-lowering medication (Figure III in the Data Supplement), or after additional adjustment for salt intake (Figure IV in the Data Supplement). Generalized additive model analyses identified evidence (*P*<5×10^–4^) of smooth terms of all analyzed blood cell counts on SBP, DBP, and PP. In particular, count of monocytes and neutrophils was associated with SBP in a dose-dependent manner (Figure V in the Data Supplement), while positive effects of lymphocytes on SBP could be observed above the 2nd quintile of lymphocyte count (Figure V in the Data Supplement). In addition, generalized additive model analyses confirmed protective effect of higher eosinophil count on all BP indices (Figure V in the Data Supplement).

### MR Analysis of White Blood Cell Counts and Blood Pressure Indices

We next aimed to investigate causal relationships between major white blood cell counts and BP indices using 121, 87, 146 (147 for DBP analysis), 126 (127 for DBP analysis), and 50 uncorrelated SNPs used as IVs for analysis of total lymphocyte, neutrophil, monocyte, eosinophil, and basophil counts, respectively (see Excel file in the Data Supplement for a list of all SNPs used as IVs).

**Figure 2.**
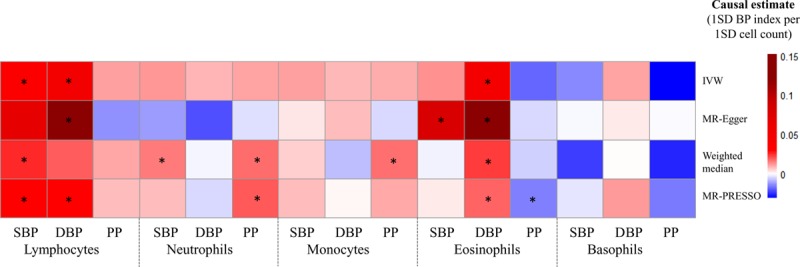
**Mendelian randomization (MR) analyses testing the effects of 5 white blood cell subpopulation counts on systolic blood pressure (SBP), diastolic blood pressure (DBP), and pulse pressure (PP).** Results obtained using 4 MR methods (inverse-variance weighted [IVW], Mendelian randomization-Egger [MR-Egger], weighted median, and MR-PRESSO [Mendelian Randomization Pleiotropy Residual Sum and Outlier]) are presented as a heat map representing causal estimates (1 SD of BP index per 1 SD of cell count). BP indicates blood pressure. *False discovery rate *P*<0.05 for a particular MR approach.

We identified positive, potential causal relationships between lymphocyte count with SBP and DBP, concordant in all analytic approaches (Figure 2 and Table IV in the Data Supplement). In particular, after false discovery rate correction for multiple testing, IVW and MR-PRESSO methods demonstrated a potential causal relationship between lymphocyte count and both BP indices (with a 1 SD genetically instrumented higher lymphocyte count leading to a 0.69 mm Hg [95% CI, 0.19–1.20] higher SBP; *P*=0.007 and 0.56 mm Hg [95% CI, 0.23–0.90] higher DBP; *P*=0.001), with similar findings when using robust MR approaches, including MR-Egger and weighted median methods (Figure 2 and Table IV in the Data Supplement). Of note, using ICBP-only data (with no participant overlap), we demonstrated consistent, positive effects between total lymphocyte count and both SBP and DBP using all 4 analytical methods (Table V in the Data Supplement).

All MR analytic approaches demonstrated a relationship between total eosinophil count and higher DBP level (Figure 2 and Table IV in the Data Supplement), and this finding was replicated using BP estimates derived from the ICBP consortium (Table V in the Data Supplement). Of note, MR-Egger found relatively strong evidence for pleiotropy (Tables IV and V in the Data Supplement), and causal effects were in the opposite direction when compared with the observational findings (Figure 1). No association concerning total neutrophil, monocyte, or basophil counts, observed in the primary analysis using UKB+ICBP BP estimates (Figure 2 and Table IV in the Data Supplement), could be replicated using ICBP-only data at a nominal threshold for significance (Table V in the Data Supplement).

**Figure 3.**
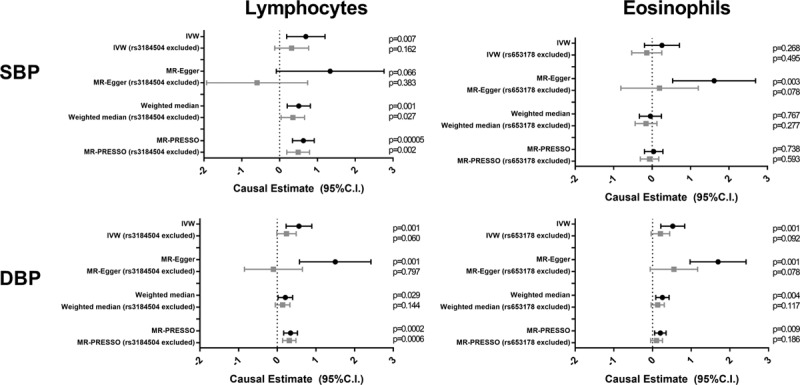
**Mendelian randomization (MR) analyses testing effect of lymphocyte or eosinophil counts on systolic blood pressure (SBP) and diastolic blood pressure (DBP) levels, before and after exclusion of a single variant from the *SH2B3/ATXN2 (SH2B adaptor protein 3/ataxin 2)* locus.** Results of 4 MR methods (inverse-variance weighted [IVW], MR-Egger, weighted median, and MR-PRESSO [Mendelian Randomization Pleiotropy Residual Sum and Outlier]) are presented as causal estimates with 95% CIs.

Inspection of scatter plots, visualizing the associations of individual SNPs (used as IVs) with BP indices and lymphocyte or eosinophil counts, as well as leave-one-out sensitivity analyses, identified a single variant in the *ATXN2*/*SH2B3* locus that influenced the IVW association (Figures VI and VII in the Data Supplement). Exclusion of these variants from MR analyses on lymphocyte count (rs3184504) or eosinophil count (rs653178) attenuated the causal estimates (Figure 3). In this scenario (ie, on excluding rs653178), the relationship of eosinophil count with SBP attenuated, while the magnitude of the positive association of lymphocyte count with SBP (weighted median and MR-PRESSO methods) or DBP (IVW and MR-PRESSO methods; Figure 3) was reduced.

### Reverse MR Analysis Assessing the Effect of BP on Blood Cell Counts

**Figure 4.**
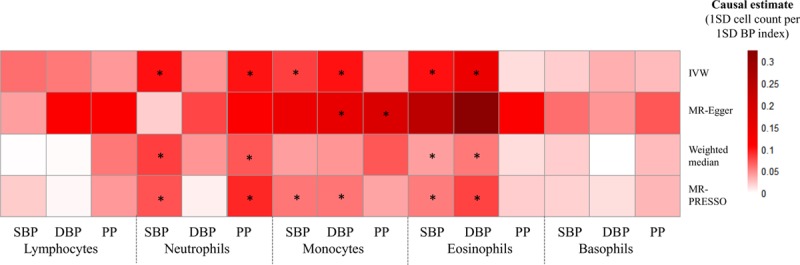
**Reverse Mendelian randomization (MR) analyses testing the effects of systolic blood pressure (SBP), diastolic blood pressure (DBP), and pulse pressure (PP) on cell counts of white blood cell subpopulations.** Results obtained using 4 MR methods (inverse-variance weighted [IVW], MR-Egger, weighted median, and MR-PRESSO [Mendelian Randomization Pleiotropy Residual Sum and Outlier]) are presented as a heat map representing causal estimates (1 SD cell count per 1 SD blood pressure [BP] index).*False discovery rate *P*<0.05 for a particular MR approach.

To further understand a source of observational associations between BP and blood cell counts, we used BP-associated variants as IVs to explore the relationship of BP traits with blood cell traits in the reverse direction. We found no evidence of association between any of the BP index and lymphocyte or basophil count (Figure 4 and Table VI in the Data Supplement; see Excel file in the Data Supplement for a list of all SNPs used as IVs). Of interest, MR analyses supported the existence of positive effects of SBP and DBP on monocyte and eosinophil count, while positive effects of SBP and PP were identified for neutrophil count (Figure 4 and Table VI in the Data Supplement). Inspection of scatter plots, visualizing the effects of all individual IVs on cell counts and BP indices, as well as leave-one-out sensitivity analyses, found that while rs3184504 SNP in *SH2B3* inflated the IVW causal estimates for neutrophil, monocyte, and eosinophil counts, the estimates remained robust to exclusion of this particular SNP (Figures VIII and IX in the Data Supplement).

### Renal Function and Heart Rate in the Context of the Circulating Lymphocyte Counts

Primary observational association and genetic analyses demonstrated concordant effect directions of lymphocyte count on SBP and DBP. Thus, we tested lymphocyte counts in the context of genetic correlation with kidney function and heart rate parameters. An evidence of positive association was found between the level of circulating lymphocytes and UACR using MR-PRESSO and IVW, but not other methods (Table VII in the Data Supplement). No association between the level of circulating lymphocytes and resting HR, increase in HR from resting level to peak exercise level, or estimated glomerular filtration rate was identified (Table VII in the Data Supplement). Inspection of scatter plots, visualizing effects of all IVs on lymphocyte count and UACR, as well as leave-one-out sensitivity analyses, found that IVW causal estimates remained robust to exclusion of any single SNP from MR analysis (Figures X and XI in the Data Supplement).

## Discussion

Using an MR approach, the present study identified a positive, potentially causal relationship between circulating blood lymphocyte counts and BP levels. This is of importance given the findings reported in a recent study that identified evidence in support of a causal relationship between circulating lymphocytes and risk of CHD.^15^ Although the genetic effect of lymphocyte count on CHD reported in previous studies was, at least partially, driven by variants in the major histocompatibility complex locus,^15^ our study provides a plausible mechanism by which lymphocytes might cause CHD, through increases in BP parameters. Of note, our MR analysis identified a potential causal relationship between total lymphocyte count and both SBP and DBP that was independent of the major histocompatibility complex region. This is of importance because SBP and DBP are recognized causal risk factors for cardiovascular disease.^34^

The MR concept has been widely used to elucidate potential causal relationships between various risk factors and disease outcomes. Using this approach, it has been shown that myeloid and lymphoid blood cell counts are causally related to several autoimmune diseases, yet not to chronic kidney disease or type 2 diabetes mellitus.^15^ MR analysis has been also used to make causal inference in the pathogenesis of hypertension. For example, lower estimated glomerular filtration rate causally influences DBP and hypertension.^35^ A recent study on urinary biomarkers in the UKB identified genetic correlation between SBP and 4 parameters reflecting renal function, including UACR.^36^ Moreover, genetic risk scores of resting HR or HR response to exercise are associated with BP,^24,25^ while genetic determinants of brachial artery diameter,^37^ associate with BP as well.^14^ These results, derived from GWAS, support a key role of kidney in the regulation of BP and suggest a tight relation between BP and function of sympathetic nervous system, controlling HR, and vasculature.

Of note, even though neutrophil count appeared to be the strongest component of white blood cells associated with SBP and DBP in an observational analysis in the UKB, we did not identify evidence of a causal effect on these BP indices in MR analysis. This may indicate confounding of the observational analysis, reverse causation, which was, at least partially, identified in the present study, or more acute effects of neutrophil count on BP that might not be captured by MR analysis. Indeed, data from the CANTOS trial (Canakinumab Anti-Inflammatory Thrombosis Outcomes Study), which investigated the use of canakinumab, an IL-1β blocker, in cardiovascular disease prevention, demonstrated that although the studied drug was profoundly neutropenic,^38,39^ it had no effect on BP,^40^ yet it should be emphasized that this was not a specific, neutrophil-targeting intervention. Inflammation may cause cardiovascular disease through a myriad of pathways, and BP is only one potential component of this.

Our data are important as they provide mechanistic context to observational associations that earlier studies have reported, indicating a relationship between peripheral blood lymphocytes and hypertension. In our previous studies, we have identified that hypertensive patients are characterized by increased central memory, Th17 as well as IFN-γ producing immunosenescent or effector CD8+ cells.^41,42^ Moreover, circulating lymphocytes have been associated with microvascular remodeling in hypertension.^43^ At the same time, we have recently observed relations between monocyte subpopulations and hypertension.^44^ Our present data, which include bidirectional assessment of genetic association between BP and blood cell counts, help to interpret these and may indicate that increases in monocytes with increasing blood pressure, are, similarly to neutrophils, most likely a consequence rather than a cause of hypertension.

The mechanism by which white blood cells, and especially lymphocytes, may cause hypertension remains unclear. Experimental animal data suggest that the effects of lymphocytes might be mediated by modulating vascular function, sympathetic outflow, and hypertension as well as renal sodium reabsorption and salt handling by antigen presenting cells.^1–3,5,29^ Using available, large-scale GWAS, we aimed to identify a target organ, whose function is causal to BP and might be affected by lymphocyte count. Our analysis of phenotypes related to kidney, and sympathetic nervous system function, demonstrated that genetically defined, higher total lymphocyte count related to a higher UACR using MR-PRESSO and IVW analyses, yet the associations were weaker using other analytic approaches. On the other hand, associations between lymphocyte count and UACR could be observed using UACR data derived from 2 different studies,^27,28^ which may support a causal link between lymphocyte count and albuminuria. Furthermore, this may indicate that lymphocytes affect BP via UACR, though it might be as well that lymphocytes affect UACR through independent causal pathways that include BP. Of note, recent studies indicate that a bidirectional association between albuminuria and BP exists.^45^ Moreover, whereas genetic liability to albuminuria associates with hypertension, stroke, and heart failure, it does not associate with chronic kidney disease,^45^ which may explain the lack of a causal relationship between lymphocyte count and estimated glomerular filtration rate in the present study. As a consequence, it has been proposed that albuminuria may have tubular origins, caused by sodium retention in the distal tubule.^46^

Other than the analyses of the genetic correlation between lymphocyte count and UACR, important biological information can be obtained while selecting genes in proximity to SNPs that associate with both lymphocyte count and BP indices in the concordant direction (Table VIII in the Data Supplement). Many genes, whose function may be affected by selected SNPs, have been investigated in the context of BP regulation in vivo, and previous studies linking genetics of hypertension to lymphocyte biology focused on *SH2B3* as key driver for hypertension (Table VIII in the Data Supplement). For example, an in vivo study demonstrated exacerbated hypertension, vascular dysfunction, and infiltration of IFNγ-producing CD8+ T cells in response to angiotensin II in *Sh2b3* knockout animals as compared with wild type mice.^47^ SNP rs3184504 is a missense mutation within *SH2B3* and leads to an R262W amino acid change in Lnk,^48^ which is a negative regulator of hematopoiesis and TNFα signaling in endothelial cells and may play a role in integrin signaling.^49^ Of interest, the *SH2B3* locus has been associated with various cardiovascular-related traits and diseases, including CHD,^50^ and its effect on blood pressure may be at least partially mediated by influencing β-2-microglobulin levels in humans.^51^ This suggests a potentially pleiotropic effect of *SH2B3* on BP level. Of importance, sensitivity analyses performed in the present study identified rs3184504 SNP in *SH2B3* as a potential pleiotropic outlier, and its exclusion led to attenuation of the MR relationships calculated by all analytic approaches. However, after exclusion of rs3184504 SNP, evidence of potential causal effect of lymphocyte count on BP was retained in IVW, weighted median or MR-PRESSO analytic approaches.

The interpretation of estimates derived from MR can be challenging.^52^ Nevertheless, MR relies on assumptions that may be untestable (eg, exclusion restriction, especially in the presence of unknown/unmeasured potential confounding).^53^ For example, horizontal pleiotropy, the phenomenon where genetic variants independently associate with traits other than the ones under investigation, can lead to confounding of the MR estimates. The susceptibility of the estimates derived from MR to such horizontal pleiotropy can be investigated through the application of sensitivity analyses that are more robust to such types of pleiotropy, as performed in the present study with MR-Egger and weighted median approaches.^16^ Although none of these methods, when used individually, entirely protects the findings from MR analyses from the influence of potential pleiotropic effects, a consistency of effect estimate derived across multiple sensitivity analyses adds confidence to the plausibility of the presence of a true underlying causal effect. Other forms of confounding in MR exist, such as population structure.^53^ In the present analysis, GWAS used in the identification of white blood cell counts and BP indices used methodology that take into account cryptic relatedness and population stratification,^14,15^ which should minimize the potential for such sources of confounding. A further vulnerability is the potential for overfitting in the context of having used the same dataset for discovery (ie, GWAS) and MR.^54^ Additionally, we used the ICBP GWAS on BP indices,^14^ characterized by no individuals overlapping with the GWAS on white blood cell counts, to confirm the lack of overfitting of the effects of genetically defined blood lymphocyte count on SBP and DBP levels.

In contrast with MR analyses, our observational results are likely to be more susceptible to errors induced by confounding or reverse causation phenomena. While the associations of white blood cell counts with BP seem to be independent of commonly used potential confounders such as sex, age, body mass index, salt/alcohol intake, or smoking habits, we cannot exclude the existence of residual confounding by variables influencing blood cell count and BP association. The reverse-causation phenomenon is, by definition, not feasible to address in the context of cross-sectional data, thus motivating the need for either prospective studies with comprehensive follow-up or approaches were reverse causation cannot be a phenomenon, such as MR approaches or randomized controlled trials.

In summary, the present study identified evidence in support of a potential causal link between elevated lymphocyte count and higher BP. The molecular mechanism of this association is possibly independent of the classical regulatory mechanisms related to kidney function or heart rate but might involve pathways related to albuminuria. Furthermore, results of reverse MR analyses may support potential causal effects of increased BP on higher levels of blood neutrophil, monocyte, and eosinophil but not lymphocyte or basophil counts.

## Acknowledgments

This research has been conducted using the UK Biobank Resource under application number 50282.

## Sources of Funding

This work was funded by the European Research Council (ERC and InflammaTENSION; ERC-CoG-726318; to T.J.G.), ERA-CVD/PLAQUEFIGHT/5/2018 and British Heart Foundation (RE/13/5/30177). M.S. is supported by the National Science Center, Poland (grant no. 2016/22/E/NZ4/00610). M.V.H. works in a unit that receives funding from the UK Medical Research Council and is supported by a British Heart Foundation Intermediate Clinical Research Fellowship (FS/18/23/33512) and the National Institute for Health Research Oxford Biomedical Research Center. M.T. is supported by British Heart Foundation grants PG/17/35/33001 and PG/19/16/34270, and Kidney Research UK grant RP_017_20180302. R.B.S. has received funding from the ERC under the European Union’s Horizon 2020 research and innovation program (grant agreement no. 648131), and German Center for Cardiovascular Research (81Z1710103). P.M. is supported by the British Heart Foundation grant PG/19/84/34771.

## Disclosures

None.

## Supplementary Material


