# Supplementary material for: White Blood Cells and Blood Pressure: A Mendelian Randomization Study
Source: Circulation. 2020 Mar 9;141(16):1307–17. doi: 10.1161/CIRCULATIONAHA.119.045102 (PMC7176352; doi:10.1161/CIRCULATIONAHA.119.045102)
Supplement: Supplementary file 1 [file cir-141-1307-s001.pdf]

SUPPLEMENTAL MATERIAL

Siedlinski et al. “White blood cells and blood pressure – a Mendelian randomization study“

Supplemental Excel File

Characteristics of SNPs used as Instrumental Variables (IVs) in Mendelian Randomization (MR) analyses

Eight sheets present data on SNPs used as IVs for 8 exposures (blood neutrophil, basophil, eosinophil, lymphocyte and monocyte count as well as SBP, DBP and PP) with corresponding data concerning outcomes tested. Data were derived from the GWAS on white blood cell counts and BP indices [1, 2]. File includes information on chromosomal location (CHR and BP), SNP rs ID, reference (REF) and alternative (ALT) alleles, frequency of the alternative allele in the GWAS on white blood cell counts (ALT_FREQ)[1], effect (Effect) and standard error (StdErr or SE) estimates for all SNPs used in MR analyses (Figures 2, 3, and 4, as well as Suppl. Tables 4 and 6 and Suppl. Figures 6, 7, 8 and 9).

Supplemental Methods

Blood pressure measurement procedure in the UK Biobank study

The participant was collected from the waiting area and was seated in a curtained office. The participant took the first part of the interview questionnaire. After completing this, the staff member informed the participant that they would now take the first of two measurements of blood pressure. The participant was asked to sit with their feet parallel to each other, toes pointing forward and soles of feet flat on the floor. There should be no restrictive clothing to impede the circulation to their left upper arm: the participant was asked to loosen or remove any restrictive clothing. The right arm was only used if the left was not practical: e.g. amputee, shunt, mastectomy, axiliary clearance). Since resting blood pressure was measured, the staff member took care not to engage the participant in conversation. A Seca tape measure was used to determine the circumference of midpoint of upper arm, and the appropriate size of blood pressure cuff was selected. The appropriate sized cuff was put on the upper arm of participant (it might, depending on size of upper arm, encircle the arm several times). The cuff was rotated so that the green marker tab indicating the centre of the cuff bladder lied over the brachial artery, which was located at the inner part of front of the elbow. The rubber inflation tubing sit over the brachial artery in line with the participant's middle finger, and the bottom of cuff sit 1-2 cm above the elbow joint. When the cuff was correctly positioned it was secured with Velcro fasteners. The participant was asked to place their arm on the desktop, so that the cuff was at about same level as their heart, and to breathe in and out slowly five times in a relaxed fashion. The rubber inflation tubing was connected to the Omron blood pressure monitor and, „Start‟ on the monitor was pressed. When the blood pressure result was displayed on monitor, the „Retrieve‟ button on the computer was selected to transfer results from the monitor and then „Accept‟ to complete the first measurement. If there was a problem with the measurement, „Reject‟ was selected and the measurement repeated. If the largest cuff size was too small for the participant, or if the electronic blood pressure monitor failed to produce a reading, a sphygmomanometer with an inflatable cuff was used in conjunction with a stethoscope. The IT system prompted the staff member to scan the sphygmomanometer by using the barcode reader before taking the measurement. After completing the first measurement, the rubber inflation tubing was disconnected from the Omron monitor, with the cuff left in place. The participant was asked to gently shake their arm, and open and close their hand a few times. A timer in the computer ensured that the second blood pressure reading could not be taken until at least 1 minute had elapsed. This rest period was used to complete the second part of the interview. The inflation tubing was

reconnected to the Omron blood pressure monitor and a second reading was made using the same procedure as before. Then the cuff was removed.

More details can be found at:
http://biobank.ndph.ox.ac.uk/showcase/showcase/docs/Bloodpressure.pdf

Supplemental Tables

Supplemental Table 1: Spearman's rho rank correlation coefficients for pairs of white blood cell counts in the UK Biobank study (n=384,721 subjects used in this study)

**Count of:**	Lymphocyte	Neutrophil	Monocyte	Eosinophil	Basophil
Lymphocyte	-	0.226	0.312	0.227	0.208
Neutrophil		-	0.322	0.135	0.204
Monocyte			-	0.255	0.190
Eosinophil				-	0.112
Basophil					-

All between blood cell counts correlations are significant at $p<2.2x10^{-16}$

Supplemental Table 2: Effect of continuously defined white blood cell counts on medication –adjusted BP indices.

	SBP			DBP			PP		
	Beta in mmHg/(cell count in 10^6cells/ml) (SE)	Stand. Beta	p	Beta in mmHg/(cell count in 10^6cells/ml) (SE)	Stand. Beta	p	Beta in mmHg/(cell count in 10^6cells/ml) (SE)	Stand. Beta	p
Lymphocyte count	0.154 (0.025)	0.0089	**$1.03x10^{-9}$**	0.062 (0.014)	0.0066	**$1.14\ x10^{-5}$**	0.092 (0.017)	0.0077	**$1.20\ x10^{-7}$**
Monocyte count	1.390 (0.105)	0.0193	**$<2\ x10^{-16}$**	0.664 (0.059)	0.0170	**$<2\ x10^{-16}$**	0.726 (0.072)	0.0147	**$<2x10^{-16}$**
Neutrophil Count	1.108 (0.022)	0.0759	**$<2\ x10^{-16}$**	0.470 (0.012)	0.0591	**$<2\ x10^{-16}$**	0.638 (0.015)	0.0637	**$<2x10^{-16}$**
Eosinophil count	-1.291 (0.221)	-0.0085	**$4.81x10^{-9}$**	-0.215 (0.124)	-0.0026	0.083	-1.076 (0.152)	-0.0104	**$1.24\ x10^{-12}$**
Basophil count	2.531 (0.587)	0.0062	**$1.62x10^{-5}$**	-0.981 (0.329)	-0.0044	**0.003**	3.511 (0.403)	0.0126	**$<2\ x10^{-16}$**

GLM models were adjusted for sex, age, age squared, BMI, smoking habits and alcohol intake frequency.

P values that surpass multiple testing using Bonferroni, (at P<0.05/15, given 15 tests performed), are depicted in bold.

Supplemental table 3: Results of quantile regression analysis in the UK Biobank

		SBP			DBP			PP		
	**Quintile**	**Estimate**	**SE**	**p val**	**Estimate**	**SE**	**p val**	**Estimate**	**SE**	**p val**
Lymphocytes	1st	-0.17620	0.10996	0.10908	-0.15480	0.06346	0.01472	-0.03324	0.07156	0.64225
	2nd	-0.14865	0.10841	0.17034	-0.06053	0.06164	0.32613	-0.07231	0.07052	0.30518
	4th	0.50656	0.11210	**0.00001**	0.22627	0.06305	**0.00033**	0.26505	0.07187	**0.00023**
	5th	1.49322	0.11229	**<0.00001**	0.75168	0.06300	**<0.00001**	0.66785	0.07584	**<0.00001**
	Cont.	0.314	0.031	**<0.00001**	0.145	0.038	**0.0001**	0.134	0.036	**0.0002**
Monocytes	1st	-1.04816	0.11828	**<0.00001**	-0.36924	0.06762	**<0.00001**	-0.58528	0.07460	**<0.00001**
	2nd	-0.46723	0.11643	**0.00006**	-0.08612	0.06673	0.19690	-0.28760	0.07463	**0.00012**
	4th	0.35220	0.11765	0.00276	0.32715	0.06782	**<0.00001**	0.10727	0.07633	0.15990
	5th	1.44514	0.11919	**<0.00001**	0.91387	0.06870	**<0.00001**	0.46807	0.07564	**<0.00001**
	Cont.	2.900	0.276	**<0.00001**	1.425	0.160	**<0.00001**	1.291	0.121	**<0.00001**
Neutrophils	1st	-2.44121	0.10638	**<0.00001**	-1.12897	0.06360	**<0.00001**	-1.25673	0.07164	**<0.00001**
	2nd	-1.03193	0.11004	**<0.00001**	-0.47699	0.06299	**<0.00001**	-0.59377	0.07195	**<0.00001**
	4th	1.03264	0.11432	**<0.00001**	0.48082	0.06427	**<0.00001**	0.41165	0.07457	**<0.00001**
	5th	2.36738	0.11625	**<0.00001**	1.00502	0.06661	**<0.00001**	1.21978	0.07638	**<0.00001**
	Cont.	1.128	0.027	**<0.00001**	0.504	0.015	**<0.00001**	0.583	0.017	**<0.00001**
Eosinophils	1st	0.83472	0.11054	**<0.00001**	0.33160	0.06283	**<0.00001**	0.47760	0.07322	**<0.00001**
	2nd	0.06778	0.11528	0.55658	0.26531	0.06582	**0.00006**	-0.34022	0.07419	**<0.00001**
	4th	-0.06264	0.11028	0.57005	0.11933	0.06332	0.05950	-0.22377	0.07280	0.00211
	5th	0.01241	0.11048	0.91057	0.12108	0.06454	0.06064	-0.22162	0.07231	0.00218
	Cont.	-1.107	0.235	**<0.00001**	-0.291	0.146	0.046	-0.831	0.158	**<0.00001**
Basophils	1st	-0.16347	0.09217	0.07615	0.32883	0.05384	**<0.00001**	-0.52842	0.06042	**<0.00001**
	2nd	-0.29741	0.13306	0.02541	-0.26554	0.07340	**0.00030**	-0.02692	0.09128	0.76805
	4th	0.55043	0.10991	**<0.00001**	0.16067	0.06300	0.01077	0.27699	0.07309	**0.00015**
	5th	0.33632	0.10764	0.00178	0.13604	0.06016	0.02375	0.21809	0.06851	0.00146
	Cont.	1.975	0.593	**0.0009**	-1.047	0.403	0.009	2.826	0.503	**<0.00001**

Median-oriented estimates are given in mmHg units relative to the 3rd quintile of certain cell type count. Analyses were adjusted for sex, age, age squared, BMI, smoking status and alcohol intake frequency. P values significant after Bonferroni correction for multiple testing (150 tests i.e. 5 types of blood cell counts x 3 BP indices x 10 between-quintile differences and 15 tests for continuously defined white blood cell counts) are depicted in bold.

Cont. = estimates concerning continuously defined count of particular white blood cell count

Supplemental table 4: MR analyses testing effects of counts of white blood cells subpopulations on blood pressure-related traits

		No of IVs	IVW		MR-Egger				Weighted median		MR-PRESSO		
			CE (SE)	p	CE (SE)	p	CE_{int} (SE_{int})	p_{int}	CE (SE)	p	$No_{outl.}$	CE (SD)	p
**Lymphocytes (1-SD higher)**	**SBP (mmHg)**	121	0.694 (0.259)	**0.007**	1.338 (0.727)	0.066	-0.026 (0.027)	0.344	0.506 (0.156)	**0.001**	19	0.628 (0.147)	**0.00005**
	**DBP (mmHg)**	121	0.563 (0.170)	**0.001**	1.500 (0.471)	**0.001**	-0.038 (0.018)	0.034	0.211 (0.096)	0.029	14	0.347 (0.091)	**0.0002**
	**PP (mmHg)**	121	0.155 (0.144)	0.282	-0.192 (0.404)	0.635	0.014 (0.015)	0.359	0.146 (0.104)	0.161	14	0.107 (0.099)	0.283
**Neutrophils (1-SD higher)**	**SBP (mmHg)**	87	0.241 (0.182)	0.186	-0.245 (0.803)	0.760	0.022 (0.030)	0.459	0.308 (0.117)	**0.009**	17	0.149 (0.146)	0.312
	**DBP (mmHg)**	87	0.094 (0.120)	0.433	-0.237 (0.555)	0.670	0.012 (0.021)	0.555	-0.017 (0.066)	0.793	15	-0.055 (0.096)	0.569
	**PP (mmHg)**	87	0.149 (0.103)	0.147	-0.059 (0.433)	0.892	0.011 (0.016)	0.479	0.240 (0.080)	**0.003**	15	0.275 (0.096)	**0.006**
**Monocytes (1-SD higher)**	**SBP (mmHg)**	146	0.219 (0.192)	0.256	0.052 (0.403)	0.897	0.009 (0.019)	0.639	0.107 (0.120)	0.372	22	0.154 (0.099)	0.121
	**DBP (mmHg)**	147	0.087 (0.127)	0.495	0.086 (0.267)	0.746	2.8e-05 (0.012)	0.998	-0.091 (0.069)	0.185	18	0.008 (0.061)	0.900
	**PP (mmHg)**	146	0.131 (0.109)	0.229	-0.069 (0.227)	0.763	0.010 (0.010)	0.318	0.238 (0.080)	**0.003**	19	0.138 (0.069)	0.047
**Eosinophils (1-SD higher)**	**SBP (mmHg)**	126	0.258 (0.232)	0.268	1.614 (0.551)	**0.003**	-0.062 (0.023)	0.007	-0.043 (0.146)	0.767	17	0.041 (0.123)	0.738
	**DBP (mmHg)**	127	0.523 (0.158)	**0.001**	1.695 (0.369)	**<0.001**	-0.053 (0.015)	**<0.001**	0.257 (0.088)	**0.004**	21	0.203 (0.076)	**0.009**
	**PP (mmHg)**	126	-0.265 (0.134)	0.049	-0.072 (0.327)	0.826	-0.009 (0.014)	0.518	-0.086 (0.099)	0.384	13	-0.221 (0.084)	**0.009**
**Basophils (1-SD higher)**	**SBP (mmHg)**	50	-0.291 (0.505)	0.564	-0.027 1.266)	0.983	-0.010 (0.044)	0.820	-0.476 (0.238)	0.050	8	-0.071 (0.201)	0.724
	**DBP (mmHg)**	50	0.117 (0.358)	0.744	0.022 (0.897)	0.981	0.004 (0.031)	0.908	-0.002 (0.133)	0.988	7	0.130 (0.111)	0.242
	**PP (mmHg)**	50	-0.441 (0.264)	0.095	-0.011 (0.657)	0.986	-0.016 (0.023)	0.475	-0.377 (0.166)	0.024	6	-0.228 (0.136)	0.100

CE=causal estimate, SE=standard error, int=intercept, IVW=inverse-variance weighted, IVs=instrumental variables, No_{outl}=number of outlier IVs excluded

P values significant at FDR of 0.05 are depicted in bold.

Supplemental table 5: Results of Mendelian Randomization analyses testing causal effect of blood cell counts on BP indices in the ICBP GWAS

		No of	IVW		MR-Egger				Weighted median		MR-PRESSO		
		IVs	CE (SE)	p	CE (SE)	p	CE_{int} (SE_{int})	p_{int}	CE (SE)	p	$No_{outl.}$	CE (SD)	p
**Lymphocytes (1-SD higher)**	**SBP (mmHg)**	121	0.657 (0.268)	0.014	1.629 (0.752)	0.030	-0.039 (0.028)	0.167	0.465 (0.230)	0.043	8	0.466 (0.195)	0.019
	**DBP (mmHg)**	121	0.514 (0.168)	**0.002**	1.398 (0.468)	**0.003**	-0.035 (0.018)	0.043	0.323 (0.134)	0.016	9	0.409 (0.109)	**0.0003**
	**PP (mmHg)**	121	0.195 (0.154)	0.206	0.164 (0.436)	0.707	0.001 (0.016)	0.939	0.097 (0.150)	0.517	7	0.078 (0.124)	0.528
**Neutrophils (1-SD higher)**	**SBP (mmHg)**	87	0.120 (0.339)	0.725	-0.553 (759)	0.466	0.028 (0.028)	0.322	-0.327 (0.261)	0.210	7	-0.094 (0.188)	0.617
	**DBP (mmHg)**	87	-0.019 (0.230)	0.933	-0.429 (0.517)	0.406	0.017 (0.019)	0.376	-0.344 (0.158)	0.030	8	-0.199 (0.125)	0.114
	**PP (mmHg)**	87	0.104 (0.175)	0.554	-0.260 (0.392)	0.506	0.015 (0.015)	0.299	0.030 (0.185)	0.870	5	0.109 (0.129)	0.403
**Monocytes (1-SD higher)**	**SBP (mmHg)**	146	0.120 (0.187)	0.521	-0.156 (0.391)	0.690	0.015 (0.018)	0.421	-0.128 (0.173)	0.461	8	0.040 (0.131)	0.761
	**DBP (mmHg)**	147	0.042 (0.119)	0.724	0.040 (0.249)	0.873	0.0001 (0.011)	0.992	-0.003 (0.100)	0.979	7	-0.040 (0.078)	0.609
	**PP (mmHg)**	146	0.067 (0.108)	0.536	-0.267 (0.223)	0.232	0.018 (0.010)	0.089	-0.095 (0.117)	0.416	5	0.029 (0.093)	0.757
**Eosinophils (1-SD higher)**	**SBP (mmHg)**	126	0.310 (0.227)	0.173	1.861 (0.533)	**0.0004**	-0.070 (0.022)	**0.001**	-0.020 (0.208)	0.925	5	0.022 (0.151)	0.886
	**DBP (mmHg)**	127	0.483 (0.154)	**0.002**	1.608 (0.360)	**8e-06**	-0.051 (0.015)	**0.001**	0.242 (0.125)	0.054	9	0.161 (0.094)	0.088
	**PP (mmHg)**	126	-0.153 (0.126)	0.225	0.246 (0.305)	0.420	-0.018 (0.013)	0.151	-0.187 (0.135)	0.165	5	-0.160 (0.104)	0.125
**Basophils (1-SD higher)**	**SBP (mmHg)**	50	-0.471 (0.506)	0.353	-0.374 (1.266)	0.767	-0.004 (0.044)	0.934	-0.455 (0.351)	0.195	6	-0.653 (0.237)	0.009
	**DBP (mmHg)**	50	-0.120 (0.315)	0.703	-0.216 (0.788)	0.784	0.004 (0.027)	0.894	-0.270 (0.200)	0.178	2	-0.268 (0.152)	0.083
	**PP (mmHg)**	50	-0.488 (0.265)	0.091	-0.090 (0.660)	0.892	-0.014 (0.023)	0.553	-0.323 (0.237)	0.173	5	-0.243 (0.183)	0.191

CE=causal estimate, SE=standard error, int=intercept, IVW=inverse-variance weighted, IVs=instrumental variables, No_{outl}=number of outlier IVs excluded

P values significant at FDR of 0.05 are depicted in bold.

Supplemental table 6: Results of reverse Mendelian Randomization analyses testing causal effect of BP indices on blood cell counts

		No of IVs	IVW		MR-Egger				Weighted median		MR-PRESSO		
			CE (SE)	p	CE (SE)	p	CE_{int} (SE_{int})	p_{int}	CE (SE)	p	$No_{outl.}$	CE (SD)	p
**SBP (mmHg)**	**Lymphocytes (1-SD higher)**	485	0.003 (0.001)	0.051	0.002 (0.004)	0.620	0.0003 (0.001)	0.795	1.06e-05 (0.001)	0.993	20	0.00106 (0.000989)	0.285
**DBP (mmHg)**		474	0.005 (0.003)	0.031	0.010 (0.006)	0.106	-0.001 (0.001)	0.405	7.9e-05 (0.002)	0.971	27	0.000286 (0.00171)	0.867
**PP (mmHg)**		399	0.003 (0.002)	0.194	0.008 (0.006)	0.164	-0.001 (0.001)	0.322	0.004 (0.002)	0.053	12	0.00297 (0.00168)	0.078
**SBP (mmHg)**	**Neutrophils (1-SD higher)**	485	0.005 (0.001)	**5.5e-05**	0.001 (0.003)	0.654	0.001 (0.001)	0.245	0.004 (0.001)	**0.003**	24	0.00360 (0.00096)	**1.9e-04**
**DBP (mmHg)**		474	0.004 (0.002)	0.076	0.007 (0.005)	0.191	-0.001 (0.001)	0.512	0.004 (0.002)	0.071	27	0.000522 (0.00165)	0.752
**PP (mmHg)**		399	0.007 (0.002)	**0.0002**	0.008 (0.005)	0.122	-0.0002 (0.001)	0.842	0.005 (0.002)	**0.006**	19	0.00641 (0.001596)	**7.21e-05**
**SBP (mmHg)**	**Monocytes (1-SD higher)**	485	0.004 (0.001)	**0.0004**	0.007 (0.003)	0.037	-0.001 (0.001)	0.421	0.002 (0.001)	0.041	23	0.00285 (0.00969)	**0.0034**
**DBP (mmHg)**		474	0.009 (0.002)	**1.26e-05**	0.014 (0.005)	**0.007**	-0.001 (0.001)	0.299	0.004 (0.002)	0.051	21	0.00521 (0.00161)	**0.00133**
**PP (mmHg)**		399	0.003 (0.002)	0.076	0.013 (0.005)	**0.013**	-0.002 (0.001)	0.050	0.005 (0.002)	0.014	22	0.00267 (0.00156)	0.087
**SBP (mmHg)**	**Eosinophils (1-SD higher)**	485	0.005 (0.001)	**0.001**	0.012 (0.003)	**0.001**	-0.002 (0.001)	0.024	0.002 (0.001)	0.095	13	0.002739 (0.000920)	**0.00306**
**DBP (mmHg)**		474	0.013 (0.003)	**1.58e-07**	0.029 (0.006)	**5.85e-06**	-0.003 (0.001)	0.008	0.005 (0.002)	0.017	20	0.00714 (0.00157)	**7.12e-06**
**PP (mmHg)**		399	0.001 (0.002)	0.500	0.008 (0.005)	0.075	-0.002 (0.001)	0.099	0.001 (0.002)	0.696	5	0.00145 (0.00151)	0.339

**SBP (mmHg)**	**Basophils (1-SD higher)**	485	0.001 (0.001)	0.220	0.003 (0.002)	0.135	-0.001 (0.001)	0.266	0.001 (0.001)	0.297	8	0.0009634 (0.000777)	0.216
**DBP (mmHg)**		474	0.003 (0.002)	0.065	0.004 (0.004)	0.244	-0.0003 (0.001)	0.637	-5.95e-05 (0.002)	0.974	10	0.00120 (0.00134)	0.371
**PP (mmHg)**		399	0.002 (0.001)	0.245	0.005 (0.004)	0.197	-0.001 (0.001)	0.350	0.002 (0.002)	0.162	6	0.00211 (0.001268)	0.096

CE=causal estimate, SE=standard error, int=intercept, IVW=inverse-variance weighted, IVs=instrumental variables, No_{outl}=number of outlier IVs excluded

P values significant at FDR of 0.05 are depicted in bold.

Supplemental table 7: MR analysis on blood lymphocytes count and selected renal, cardiac and vascular function outcomes

				IVW			Weighted Median					
**Outcome**	**Study**	**Appr. N***	**No. of IVs**	**CE**	**SE**	**p**	**CE**	**SE**	**p**			
eGFR	Wuttke M et al.[3]	533 000	125	0.002	0.002	0.399	0.001	0.002	0.472			
UACR EA	Teumer A et al. [4]	547 000	123	0.023	0.009	0.011	0.015	0.009	0.117			
UACR	Teumer A et al. [5]	54 000	64	0.065	0.028	0.020	0.033	0.037	0.374			
Resting HR	Eppinga R et al.[6]	134 000	148	-0.161	0.170	0.344	0.055	0.179	0.757			
HR increase	Verweij N et al.[7]	58 000	124	0.006	0.017	0.730	-0.002	0.025	0.920			
				**MR-PRESSO outlier corrected[$]**			**MR-Egger**					
**Outcome**	**Study**	**Appr. N***	**No. of IVs**	**CE**	**SD**	**p**	**CE**	**SE**	**p**	**Intercept**	**SE intercept**	**p intercept**
eGFR	Wuttke M et al.[3]	533 000	125	0.0016	0.0014	0.258	-0.005	0.006	0.353	0.0003	0.0002	0.176
UACR EA	Teumer A et al. [4]	547 000	123	0.0199	0.0076	0.010	0.009	0.025	0.715	0.001	0.001	0.551
UACR	Teumer A et al. [5]	54 000	64	0.0569	0.0264	0.035	-0.032	0.075	0.670	0.004	0.003	0.166
Resting HR	Eppinga R et al.[6]	134 000	148	-0.0005	0.1429	0.997	0.529	0.446	0.236	-0.028	0.017	0.095
HR increase	Verweij N et al.[7]	58 000	124	0.0058	0.0169	0.730	0.033	0.044	0.456	-0.001	0.002	0.507

*average number of subjects with IVs data available in the outcome study (rounded to 1 000)

[$] Analyses excluded 12, 4, 1, 5 and 0 IVs, identified as outliers, for eGFR, UACR EA, UACR, resting HR and HR increase respectively

IV=instrumental variable, CE=causal estimate, HR=Heart rate, BUN=blood urea nitrogen level, UACR=urinary albumin to creatinine ratio, FMD=flow-mediated dilatation, eGFR= estimated Glomerular Filtration Rate; EA= European-American ancestry

Supplemental table 8: A summary of selected literature on the function genes in proximity to SNPs associated with both lymphocyte count and BP indices

SNP rsID	Type of SNP	Proximal gene(s)	Associated phenotype	Gene's function	Results of gene-focused *in vivo* studies
rs3184504	missense	*SH2B3*	SBP, DBP	Also known as lymphocyte adapter protein (LNK) regulates proliferation and differentiation of immune cells and activity of endothelial NOS [8, 9]	exacerbated hypertension, vascular dysfunction and infiltration of IFNg-producing CD8+ T cells in response to AngII in *Sh2b3* knockout animals as compared to wild type mice [10]
rs7650602	intron	*ZBTB38*	DBP	Zinc finger and BTB domain-containing protein 38 binds methylated CpGs and represses gene transcription [11]	-
rs7939778	intergenic	*ARHGAP42*	SBP, DBP	Rho GTPase activating protein 42 (also known as GRAF3) inhibits RhoA-mediated contractility in vascular smooth muscle cells [12]	exacerbated hypertension in *ARHGAP42* knockout mice as compared to wild type animals due to increased contractile responses to AngII/endothelin-1 of the isolated vessels [12]; increased susceptibility to develop DOCA-salt-mediated hypertension of *ARHGAP42* knockout mice [13]
rs9494142	intergenic	*HBS1L and MYB*	DBP	HBS1 like translational GTPase is a member of the GTP-binding elongation factor family;<br>c-MYB is a transcription factor which regulates hematopoiesis and VSMC proliferation and differentiation [14-16]	mutation in transactivation domain of c-*Myb* results in lymphopenia in mice [16]; mice homozygous for hypomorphic c-*Myb* allele have reduced peripheral/renal B-cells, decreased SBP and DBP and lowered susceptibility to DOCA-salt-mediated hypertension [17];
rs696	3' UTR	*NFKBIA*	SBP, DBP	NF-kappa-B inhibitor alpha (also known as IκBα) sequesters stress-induced transcription factor NF-κB in an inactive state in the cytoplasm; NF-κB plays crucial role in lymphocyte development/function and thus in adoptive immunity [18, 19]	endothelium-specific IκBα overexpression results in milder renal damage and reduced T cell infiltration, but has no effect on blood pressure level in hypertension as compared to wild type controls [20]; pharmacological inhibition of NF-κB signaling reduces cardiac hypertrophy in spontaneously hypertensive rats (SHRs) without effects on BP level [21]
rs9913156	intergenic	*ARRB2*	DBP	Arrestin beta 2 is a regulator of G-protein coupled receptor (GPCR) signaling [22]	no significant difference in AngII-induced SBP raise in $Apoe^{-/-}$ $Arrb2^{-/-}$ mice as compared to $Apoe^{-/-}Arrb2^{+/+}$ [23]; angiotensin II type 1 receptor (AGTR1)/Arrb2/ERK1/2 signaling axis regulates renal fibrosis [24]
rs60515486	intron	*AGBL2*	SBP, DBP	*AGBL2* gene encodes cytoplasmic carboxypeptidase 2 (CCP2) that is responsible for deglutamylation of targeted protein such as<br>α-tubulin or myosin light chain kinase (MYLK) [25]	-
rs76382185	intergenic	*S1PR1*	DBP	S1P receptor type 1 and its ligand, Sphingosine-1-Phosphate are crucial for the egress of lymphocytes from lymph nodes and for regulation of eNOS dependent vasodilation[26, 27]	EC-specific *S1pr1* knockout mice have increased basal blood pressure level, as well exacerbated hypertension in response to AngII infusion [26]; S1pr1 agonist, SEW2871, administration decreases BP of hypertensive mice [28]

rs3745621	intron	*SAE1*	DBP	SAE1 activates SUMO (1-3) proteins, which covalently bind to target proteins introducing post-translational modification called SUMOylation [29, 30]	AngII induces upregulation of SUMO-1 and its target, activating transcription factor 3 (ATF3) in the mice aorta. ATF3 SUMOylation is involved in endothelial cell dysfunction and inflammation via increasing ATF3 protein stability [31]; SUMO-1 overexpression protects against pressure overload-induced left ventricular hypertrophy [32]; SUMOylation of RORγt transcription factor by SUMO 2 is crucial for the T_H17 lymphocyte differentiation and thymocyte development [33]
rs6589939	intergenic	*UBASH3B*	DBP	Ubiquitin-associated and SH3 domain-containing protein B (UBASH3B, also termed STS-1 or TULA-2) negatively regulates signaling through T cell receptor (TCR) [34]	T cells isolated from UBASH3B and UBASH3A double knockout mice are hyper-responsive to TCR stimulation and exhibit enhanced cytokine production [34]; single knockouts also exhibit exacerbated inflammation [35]
rs12598529	intron	*ADCY9*	DBP	Adenylate cyclase 9 (AC9) catalyses the formation of cyclic AMP (cAMP) in response to beta-adrenergic signaling activation [36]; cAMP abundantly produced by AC9 in T reg cells is transferred via gap junction to responder T cells which in turn become suppressed [37, 38]	Loss of *ADCY9* results in an improved endothelial function in femoral arteries of healthy, as well as in atherosclerotic mice; loss of *ADCY9* protects mice from atherosclerosis due to reduced macrophage accumulation/proliferation in aortic wall [39]; mice with *ADCY9* deletion exhibit left ventricular diastolic dysfunction [40]
rs2073748	missense	*ARVCF*	SBP	Armadillo repeat protein deleted in velo-cardio-facial syndrome binds with cadherins forming adherens junctions [41]; ARVCF localized to the nucleus regulates alternative splicing [42]	ARVCF is required for vertebrate embryogenesis and kidney development [43, 44]
rs1260326	missense	*GCKR*	SBP	Glucokinase regulatory protein (GCKR, also known as GKRP) is a liver-specific protein that binds and inactivates glucokinase (GCK), a key enzyme in glucose metabolism [45]	*GCKR* null mice are characterized by normal blood glucose level, however, following glucose injection, demonstrate impaired glucose clearance [46]; *GCKR* knockout mice (representing Maturity Onset Diabetes of the Young 2 (MODY2) model) are characterized by increased kidney damage [47]

Presented SNPs were previously associated with lymphocyte count at $p<5x10^{-8}$ [1] and with SBP and/or DBP [2] in the same direction at Bonferroni-corrected p value threshold given 121 SNPs tested ($p<4.1x10^{-4}$) in Mendelian Randomization analysis on lymphocyte count and BP indices in the current study.

Supplemental Figures

Supplemental figure 1: Distribution of BP indices in 384,721 subjects of the UK Biobank study

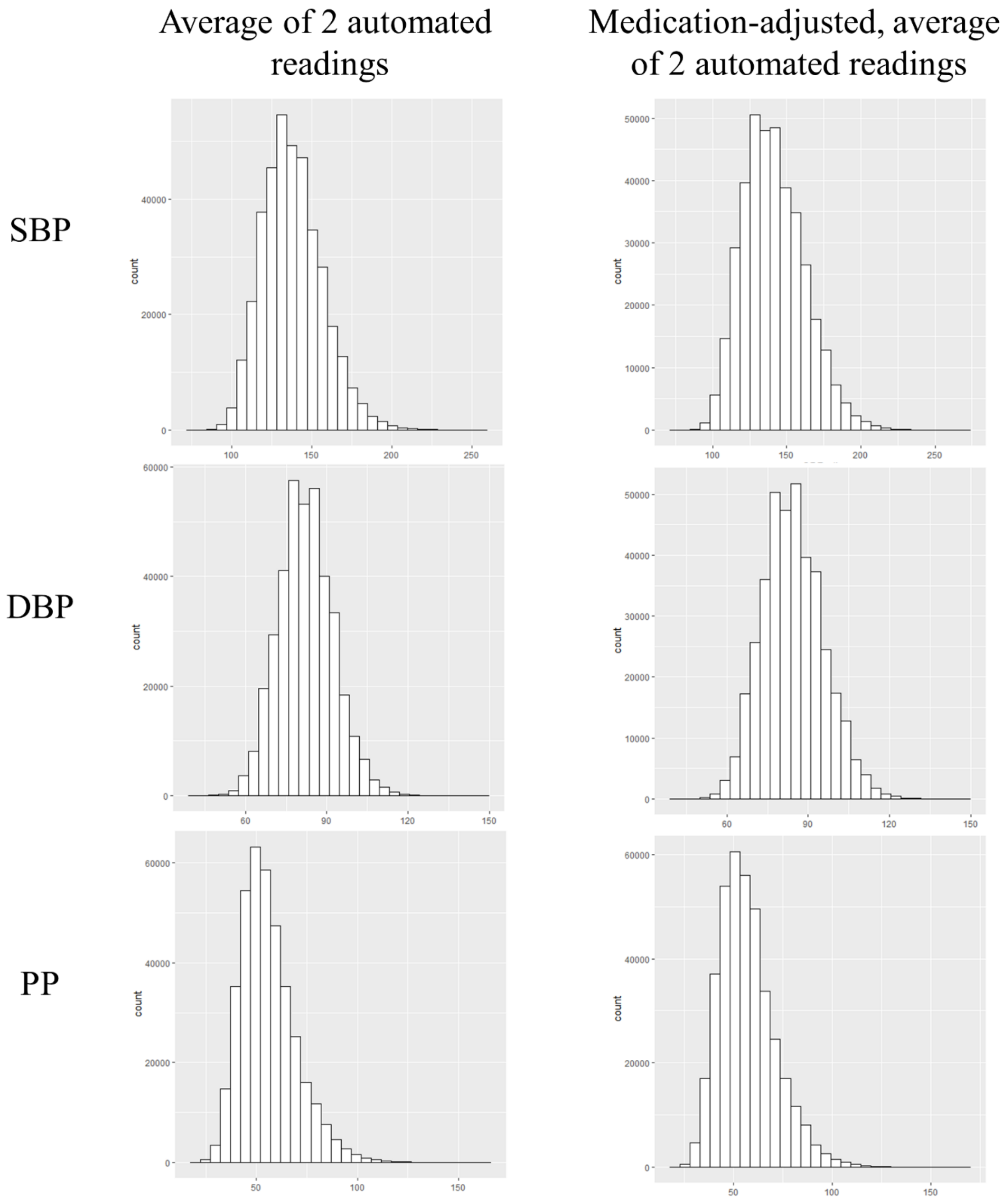

Supplemental figure 2: Distribution of white blood cell counts in 384,721 subjects of the UK Biobank study

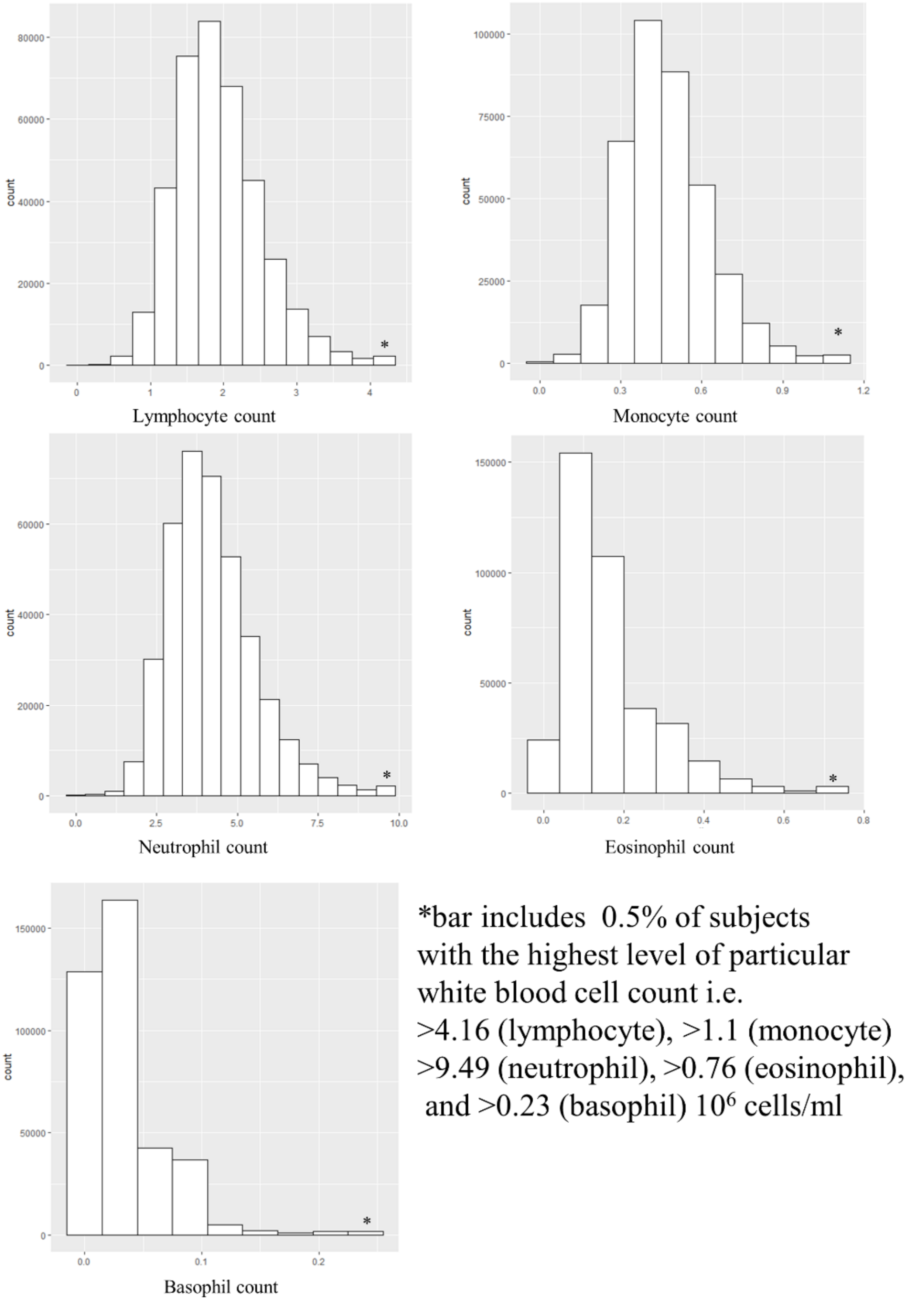

Supplemental figure 3: Level of blood pressure indices in relation to quintiles of five counts of white blood cell types after exclusion of 20.7% subjects reporting BP-lowering medication use in the UK Biobank

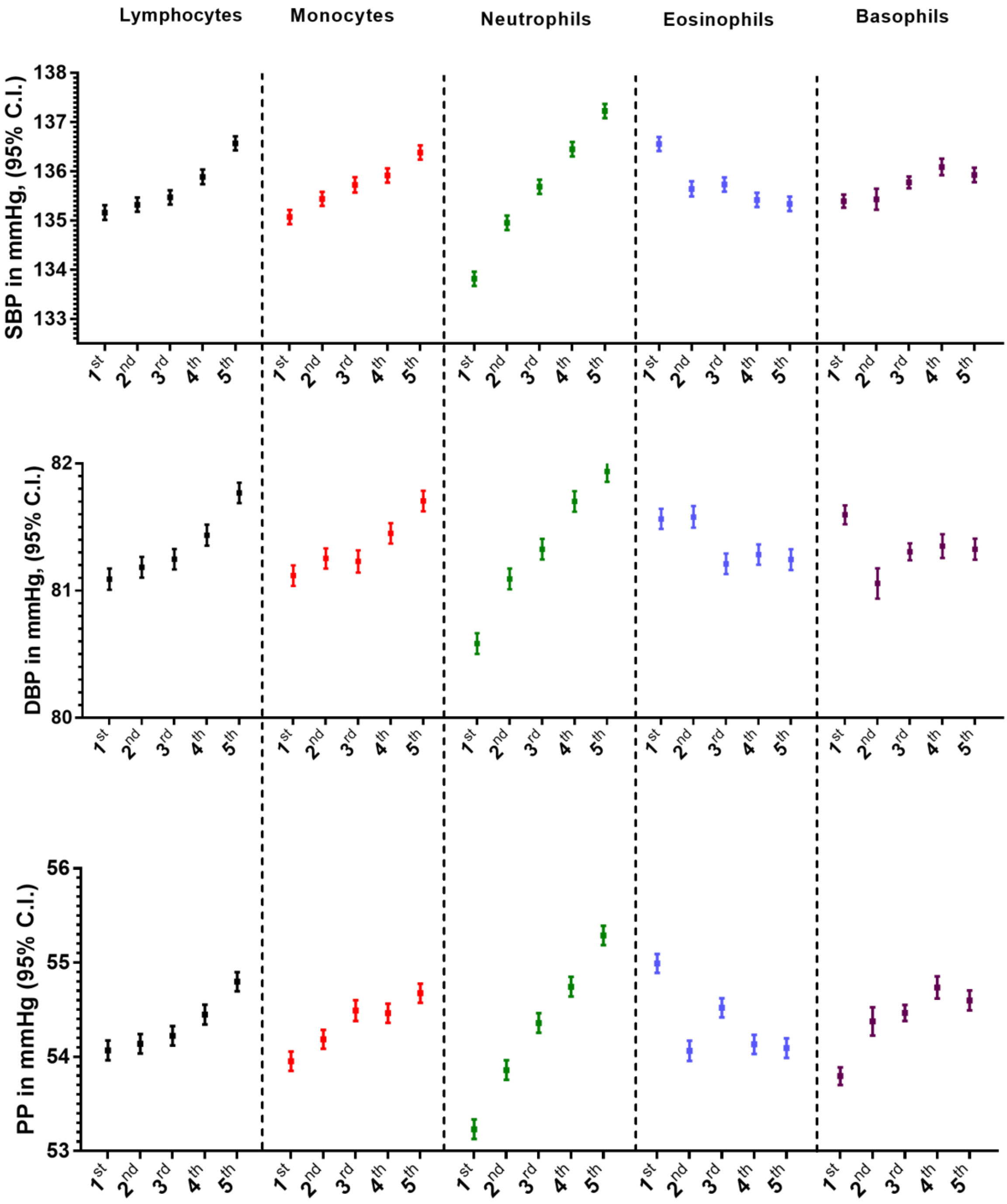

Estimated marginal means of BP indices, from GLM analysis adjusted for sex, age, age squared, BMI, smoking status and alcohol intake frequency, are presented according to quintiles of counts of white blood cell subpopulations. All ANOVA tests, assessing global between-quintiles differences in BP indices were significant at $p<10^{-12}$. Post-hoc tests revealed that all comparisons between the 1st and the 5th quintile of any cell type count with respect to any BP index were significant at Bonferroni corrected p<0.05, given 150 tests (5 types of blood cell counts x 3 BP indices x 10 between-quintile differences) performed.

Supplemental figure 4: Level of medication-adjusted, blood pressure indices in relation to quintiles of five counts of white blood cell types after additional adjustment for salt intake

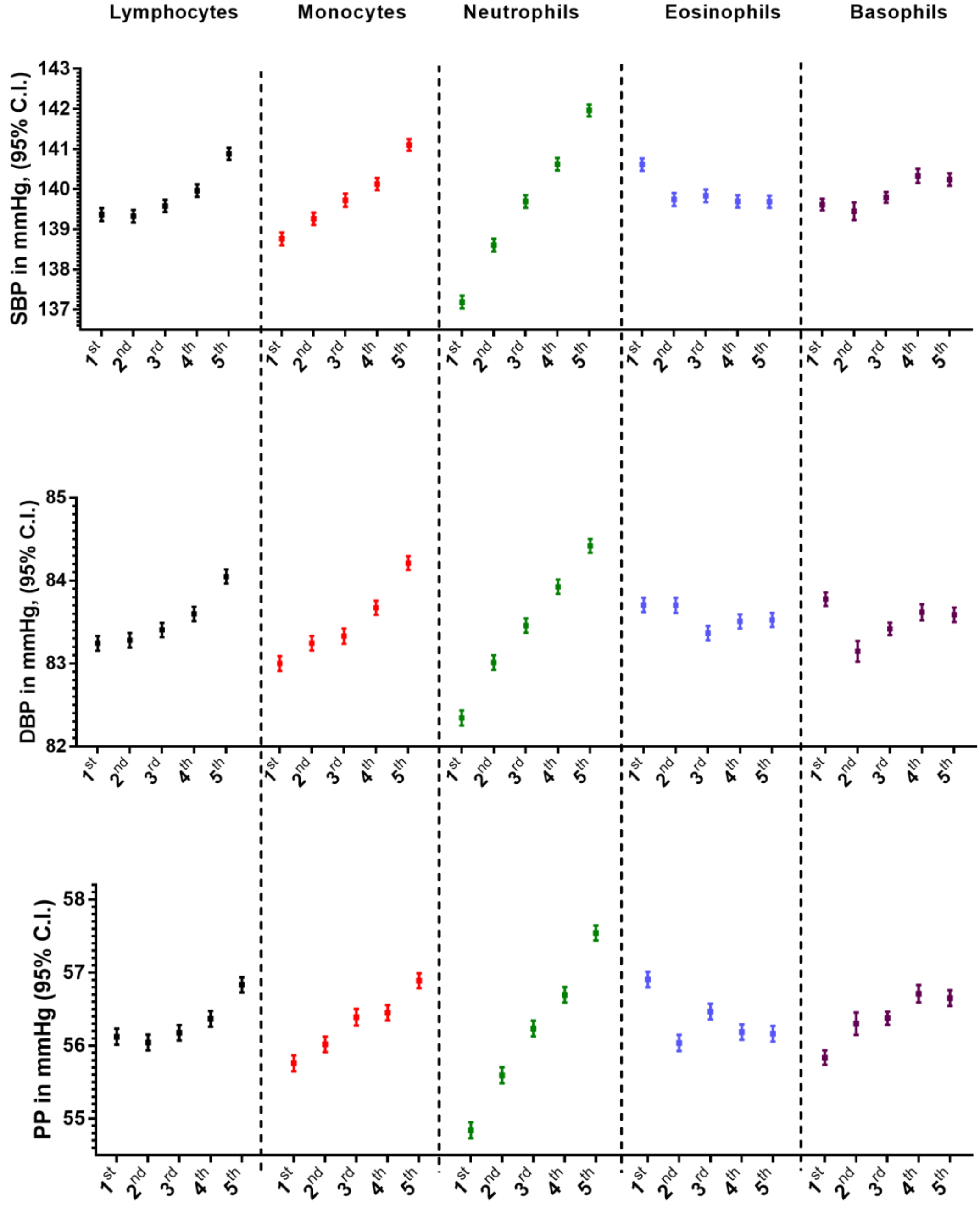

Estimated marginal means of BP indices, from GLM analysis adjusted for sex, age, age squared, BMI, smoking status, salt intake and alcohol intake frequency, are presented according to quintiles of counts of white blood cell subpopulations. All ANOVA tests, assessing global between-quintiles differences in BP indices were significant at $p<10^{-11}$. Post-hoc tests revealed that all comparisons, except for eosinophil count and DBP, between the 1st and the 5th quintile of any cell type count with respect to any BP index were significant at Bonferroni corrected $p<0.05$, given 150 tests (5 types of blood cell counts x 3 BP indices x 10 between-quintile differences) performed.

Supplemental figure 5: Partial residual plots derived from GAM analyses testing continuous level of five white blood cell types in relation to medication-adjusted blood pressure indices in the UK Biobank

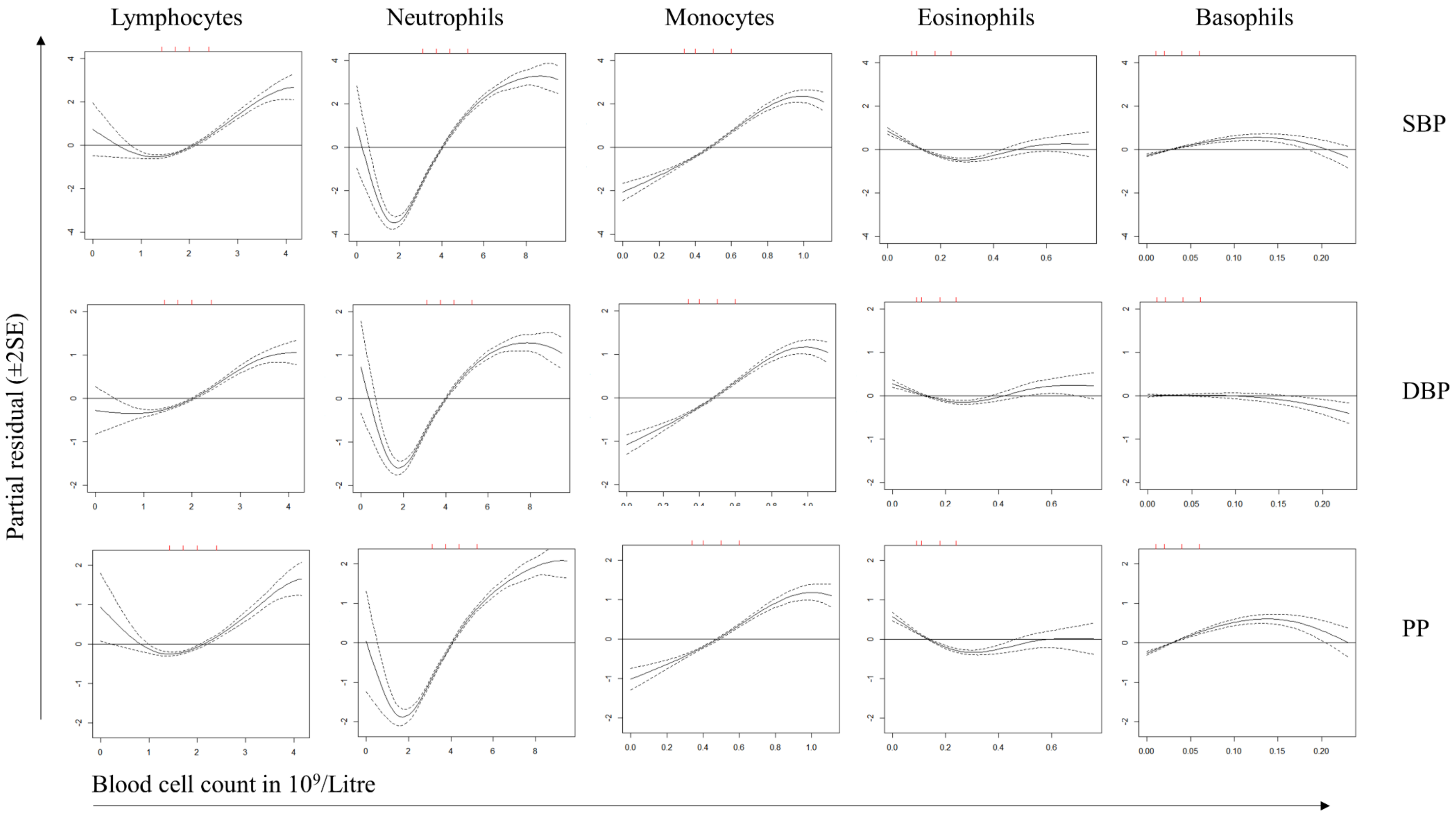

Red ticks correspond to between-quintile boundaries.

X axes were limited in order to exclude 0.5% subjects with the highest level of particular white blood cell count.

Supplemental figure 6: Scatter plots of SNPs used as IVs for lymphocyte (A,C) or eosinophil (B,D) count with SBP (A,B) or DBP (C,D).

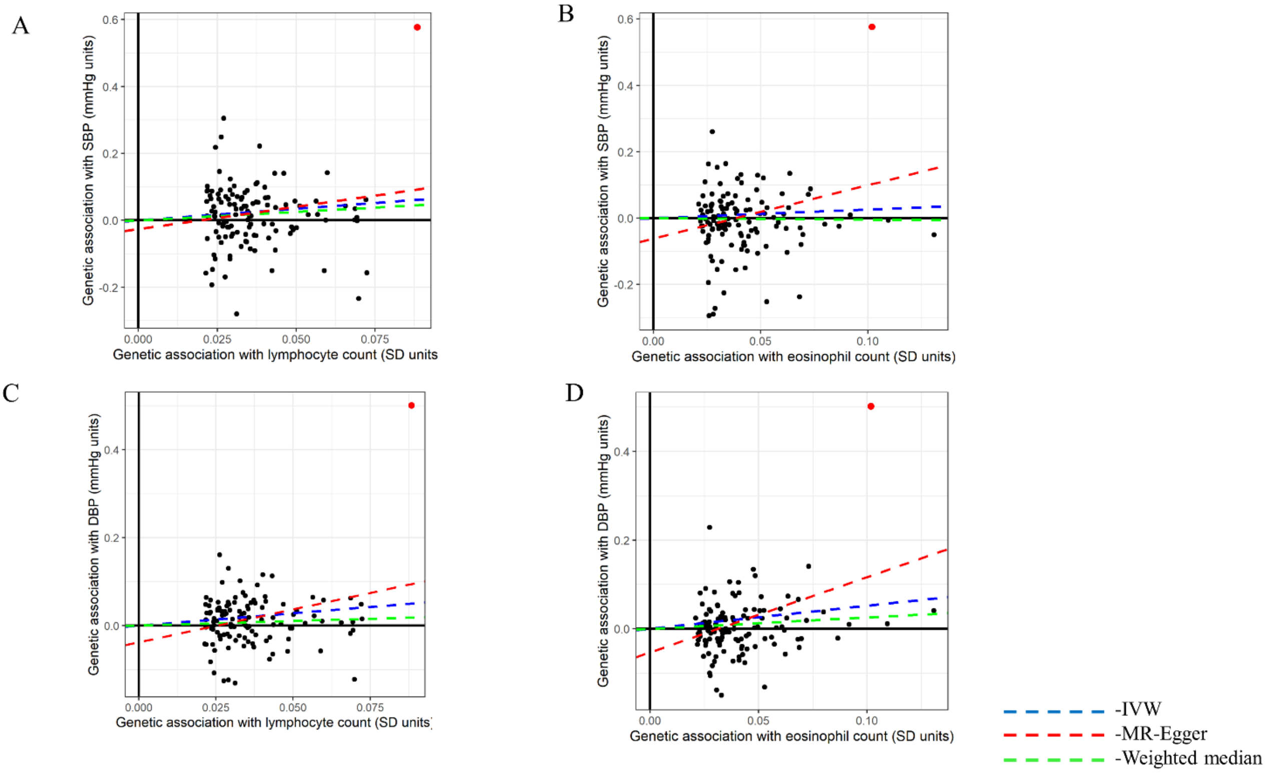

SNPs rs3184504 (A,C) and rs653178 (B,D) are depicted in red.

Supplemental figure 7: Leave-one-out plots presenting IVW causal estimates testing effect of lymphocyte or eosinophil count on SBP/DBP level

Exposure:	Lymphocytes	Lymphocytes	Eosinophils	Eosinophils
Outcome:	SBP	DBP	SBP	DBP

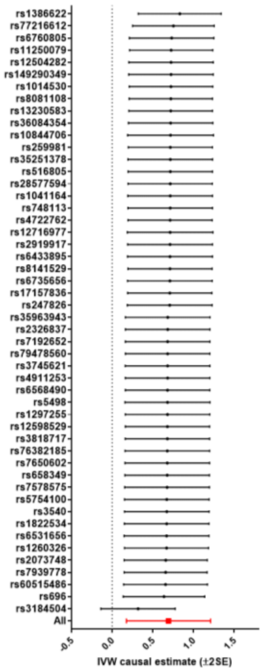

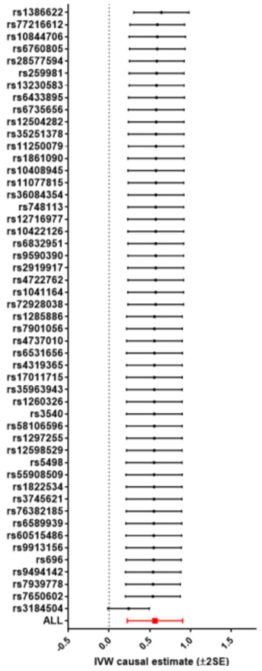

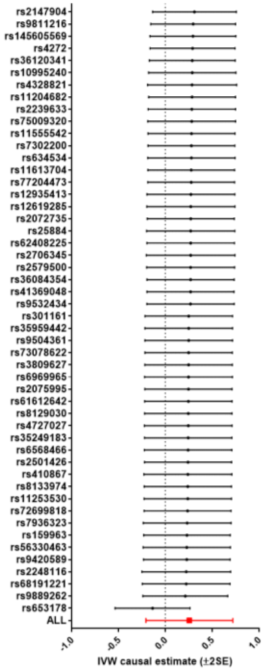

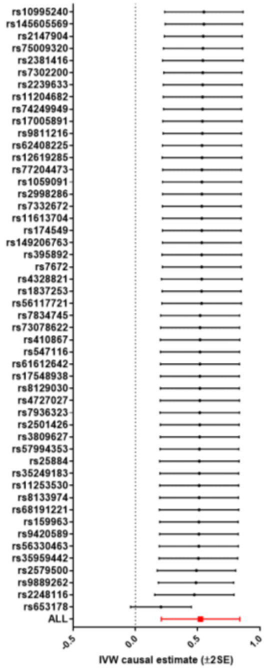

Top 25 SNPs that inflate or deflate IVW causal estimates the most are depicted.

Supplemental figure 8: Scatter plots of SNPs used as IVs for the analysis of SBP (A,B,C) and DBP (D,E,F) with neutrophil (A,D), monocyte (B,E) or eosinophil (C,F) cell count

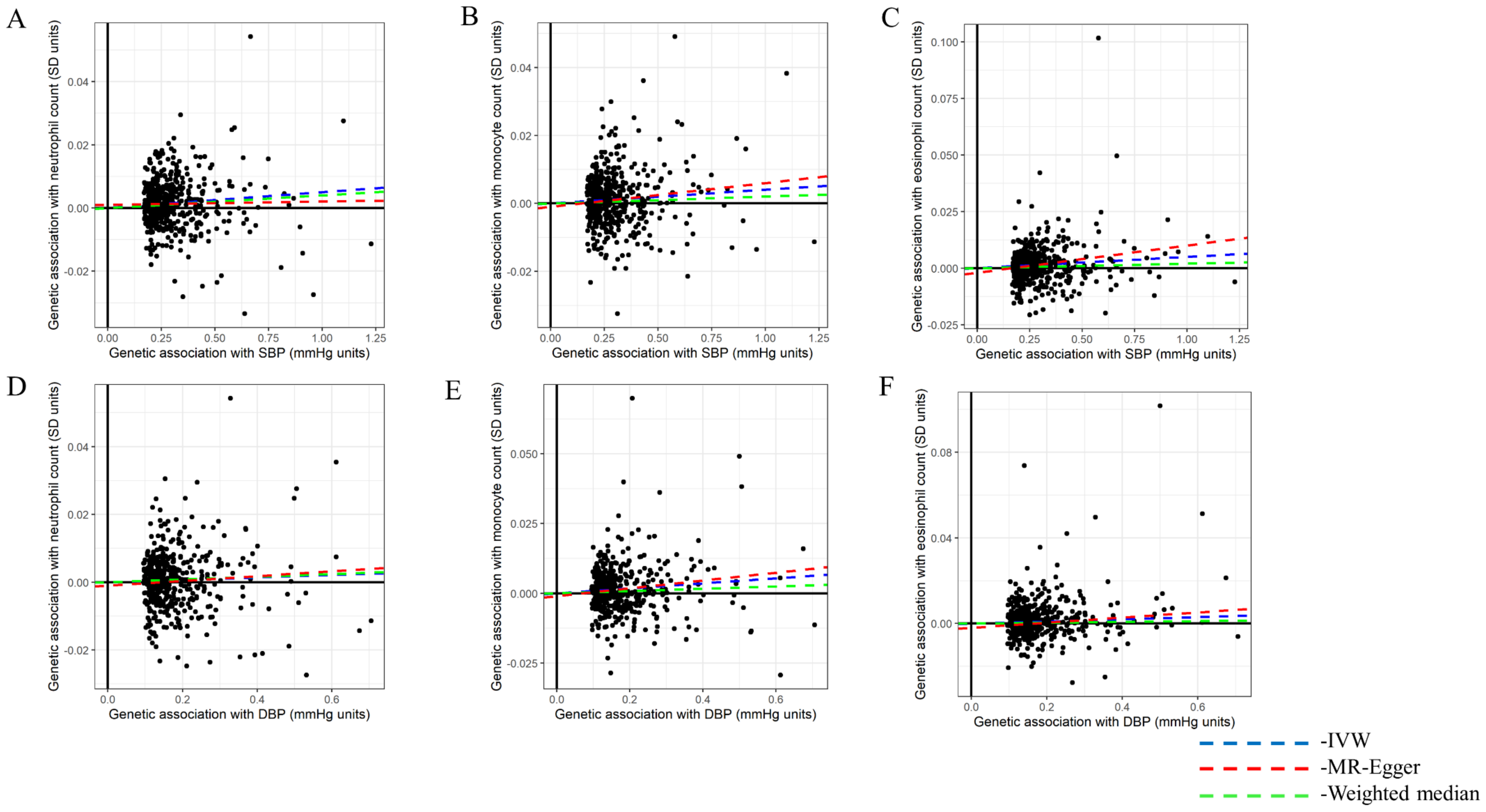

Supplemental figure 9: Leave-one-out plots presenting IVW causal estimates testing effect of SBP/DBP level on cell counts

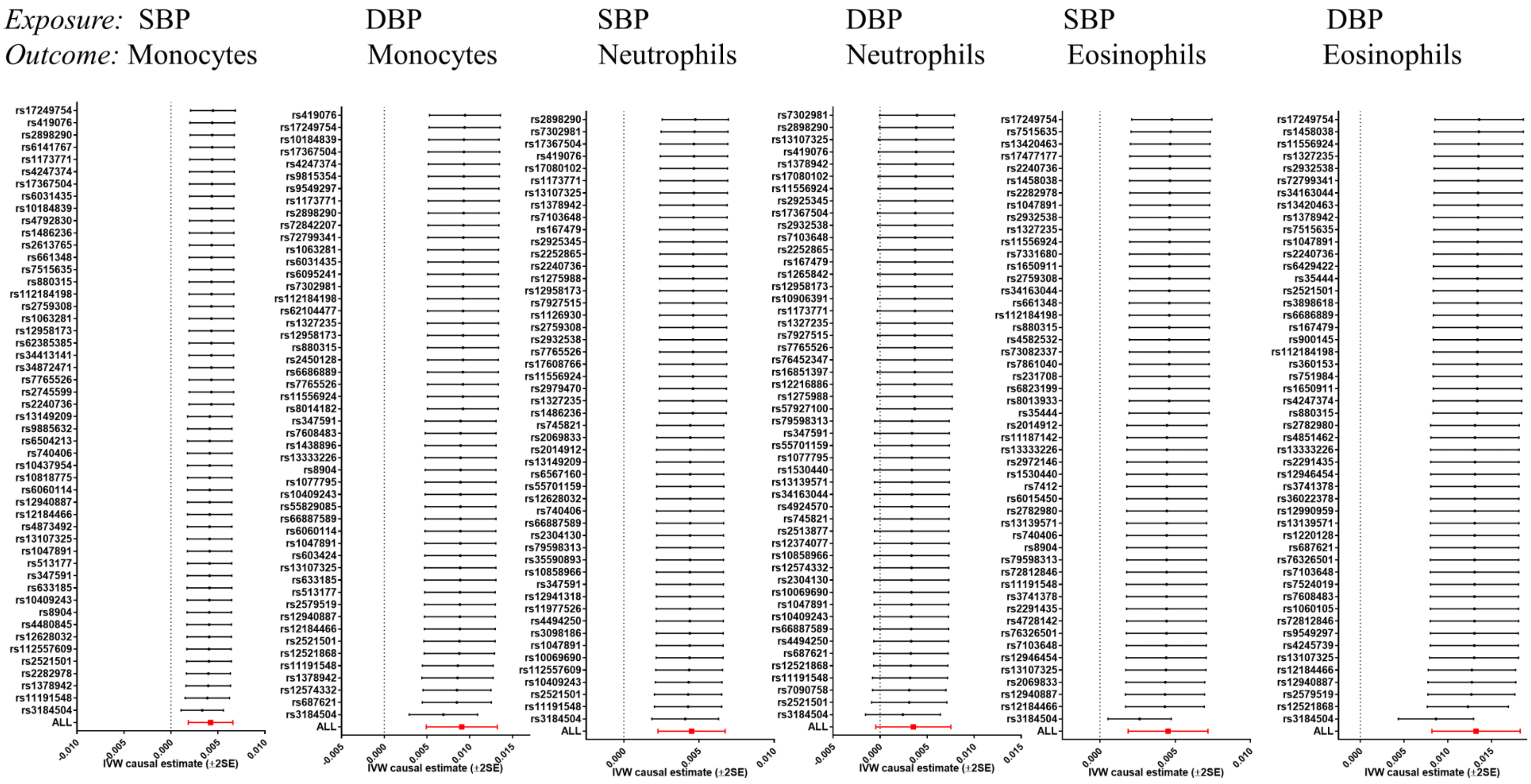

Top 25 SNPs that inflate or deflate IVW causal estimates the most are depicted.

Supplemental figure 10: Beta estimates concerning associations of SNPs, used as IVs in MR analyses, with UACR and lymphocyte count

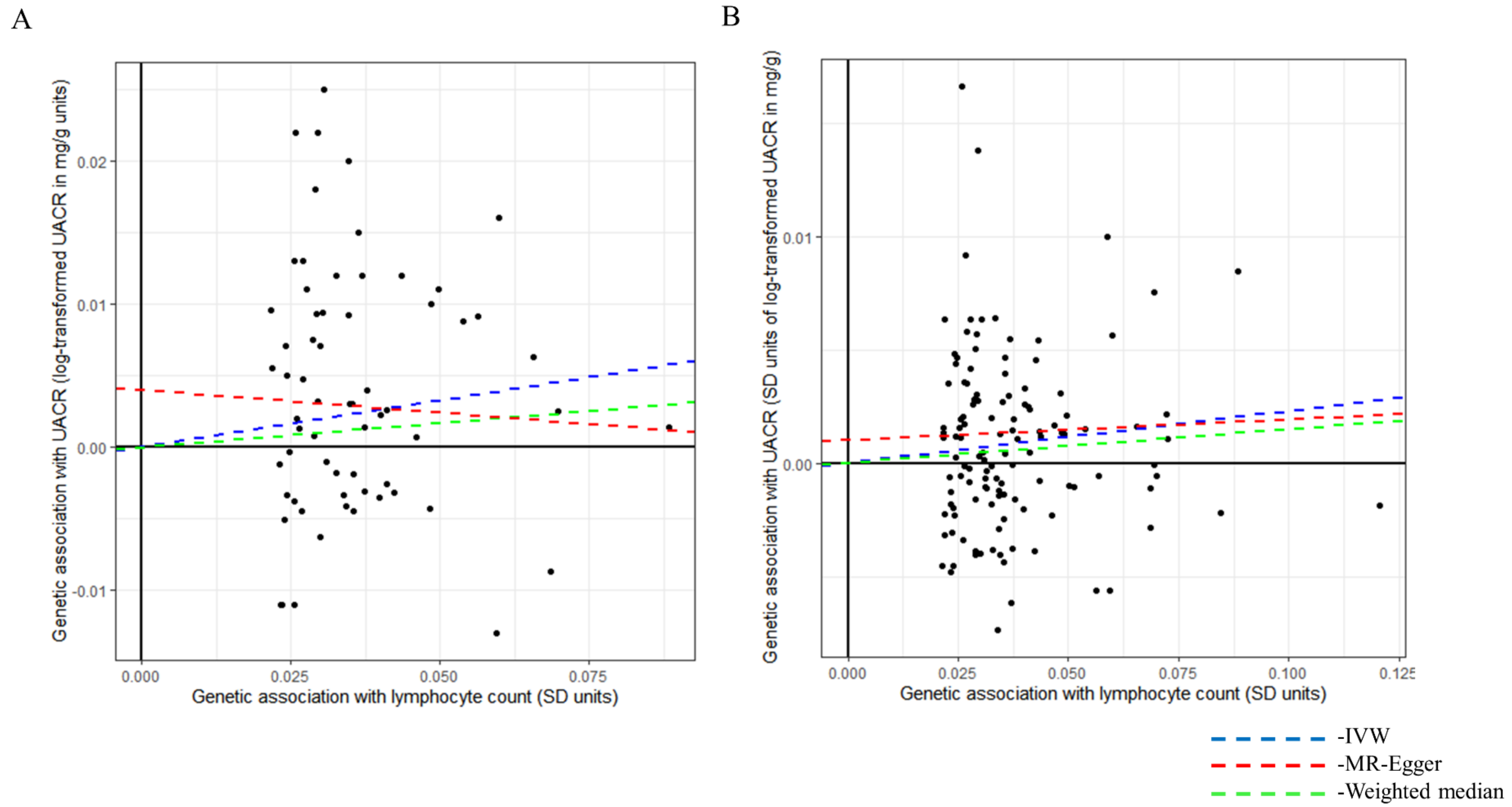

UACR SNP-specific estimates were derived from GWAS of the CKD Gen consortium (A) [5] or meta-analysis of UK Biobank and CKD Gen consortium (B) [4]

Supplemental figure 11: Leave-one-out plots presenting IVW causal estimates testing effect of lymphocyte count on UACR level

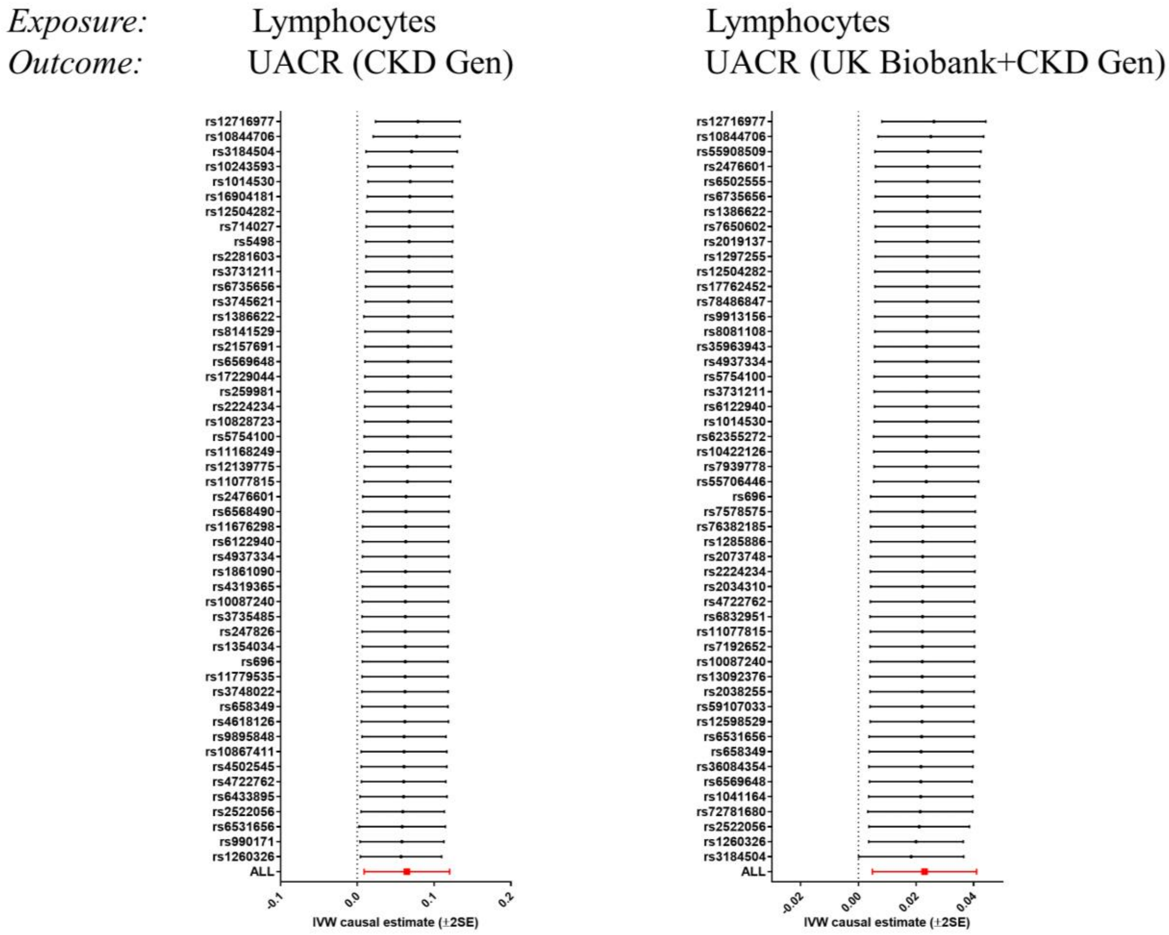

Top 25 SNPs that inflate or deflate IVW causal estimates the most are depicted.

Supplemental References

1. Astle WJ, Elding H, Jiang T, Allen D, Ruklisa D, Mann AL, Mead D, Bouman H, Riveros-Mckay F, Kostadima MA, et al. The Allelic Landscape of Human Blood Cell Trait Variation and Links to Common Complex Disease. Cell. 2016;167:1415-1429 e1419.
2. Evangelou E, Warren HR, Mosen-Ansorena D, Mifsud B, Pazoki R, Gao H, Ntritsos G, Dimou N, Cabrera CP, Karaman I, et al. Genetic analysis of over 1 million people identifies 535 new loci associated with blood pressure traits. Nat Genet. 2018;50:1412-1425.
3. Wuttke M, Li Y, Li M, Sieber KB, Feitosa MF, Gorski M, Tin A, Wang L, Chu AY, Hoppmann A, et al. A catalog of genetic loci associated with kidney function from analyses of a million individuals. Nat Genet. 2019;51:957-972.
4. Teumer A, Li Y, Ghasemi S, Prins BP, Wuttke M, Hermle T, Giri A, Sieber KB, Qiu C, Kirsten H, et al. Genome-wide association meta-analyses and fine-mapping elucidate pathways influencing albuminuria. Nat Commun. 2019;10:4130.
5. Teumer A, Tin A, Sorice R, Gorski M, Yeo NC, Chu AY, Li M, Li Y, Mijatovic V, Ko YA, et al. Genome-wide Association Studies Identify Genetic Loci Associated With Albuminuria in Diabetes. Diabetes. 2016;65:803-817.
6. Eppinga RN, Hagemeijer Y, Burgess S, Hinds DA, Stefansson K, Gudbjartsson DF, van Veldhuisen DJ, Munroe PB, Verweij N and van der Harst P. Identification of genomic loci associated with resting heart rate and shared genetic predictors with all-cause mortality. Nat Genet. 2016;48:1557-1563.
7. Verweij N, van de Vegte YJ and van der Harst P. Genetic study links components of the autonomous nervous system to heart-rate profile during exercise. Nat Commun. 2018;9:898.
8. Blass G, Mattson DL and Staruschenko A. The function of SH2B3 (LNK) in the kidney. Am J Physiol Renal Physiol. 2016;311:F682-F685.
9. Devalliere J and Charreau B. The adaptor Lnk (SH2B3): an emerging regulator in vascular cells and a link between immune and inflammatory signaling. Biochem Pharmacol. 2011;82:1391-1402.
10. Saleh MA, McMaster WG, Wu J, Norlander AE, Funt SA, Thabet SR, Kirabo A, Xiao L, Chen W, Itani HA, et al. Lymphocyte adaptor protein LNK deficiency exacerbates hypertension and end-organ inflammation. J Clin Invest. 2015;125:1189-1202.
11. Filion GJ, Zhenilo S, Salozhin S, Yamada D, Prokhortchouk E and Defossez PA. A family of human zinc finger proteins that bind methylated DNA and repress transcription. Mol Cell Biol. 2006;26:169-181.
12. Bai X, Lenhart KC, Bird KE, Suen AA, Rojas M, Kakoki M, Li F, Smithies O, Mack CP and Taylor JM. The smooth muscle-selective RhoGAP GRAF3 is a critical regulator of vascular tone and hypertension. Nat Commun. 2013;4:2910.
13. Bai X, Mangum KD, Dee RA, Stouffer GA, Lee CR, Oni-Orisan A, Patterson C, Schisler JC, Viera AJ, Taylor JM, et al. Blood pressure-associated polymorphism controls ARHGAP42 expression via serum response factor DNA binding. J Clin Invest. 2017;127:670-680.
14. Wang X, Angelis N and Thein SL. MYB - A regulatory factor in hematopoiesis. Gene. 2018;665:6-17.
15. Kolodziejska KM, Noyan-Ashraf MH, Nagy A, Bacon A, Frampton J, Xin HB, Kotlikoff MI and Husain M. c-Myb-dependent smooth muscle cell differentiation. Circulation research. 2008;102:554-561.
16. Sandberg ML, Sutton SE, Pletcher MT, Wiltshire T, Tarantino LM, Hogenesch JB and Cooke MP. c-Myb and p300 regulate hematopoietic stem cell proliferation and differentiation. Dev Cell. 2005;8:153-166.

17. Dingwell LS, Shikatani EA, Besla R, Levy AS, Dinh DD, Momen A, Zhang H, Afroze T, Chen MB, Chiu F, et al. B-Cell Deficiency Lowers Blood Pressure in Mice. Hypertension (Dallas, Tex : 1979). 2019;73:561-570.
18. Baeuerle PA. IkappaB-NF-kappaB structures: at the interface of inflammation control. Cell. 1998;95:729-731.
19. Oh H, Grinberg-Bleyer Y, Liao W, Maloney D, Wang P, Wu Z, Wang J, Bhatt DM, Heise N, Schmid RM, et al. An NF-kappaB Transcription-Factor-Dependent Lineage-Specific Transcriptional Program Promotes Regulatory T Cell Identity and Function. Immunity. 2017;47:450-465.e455.
20. Henke N, Schmidt-Ullrich R, Dechend R, Park JK, Qadri F, Wellner M, Obst M, Gross V, Dietz R, Luft FC, et al. Vascular endothelial cell-specific NF-kappaB suppression attenuates hypertension-induced renal damage. Circulation research. 2007;101:268-276.
21. Gupta S, Young D and Sen S. Inhibition of NF-kappaB induces regression of cardiac hypertrophy, independent of blood pressure control, in spontaneously hypertensive rats. Am J Physiol Heart Circ Physiol. 2005;289:H20-29.
22. Ma L and Pei G. Beta-arrestin signaling and regulation of transcription. Journal of cell science. 2007;120:213-218.
23. Trivedi DB, Loftin CD, Clark J, Myers P, DeGraff LM, Cheng J, Zeldin DC and Langenbach R. beta-Arrestin-2 deficiency attenuates abdominal aortic aneurysm formation in mice. Circulation research. 2013;112:1219-1229.
24. Wang Y, Huang J, Liu X, Niu Y, Zhao L, Yu Y, Zhou L, Lu L and Yu C. beta-Arrestin-biased AT1R stimulation promotes extracellular matrix synthesis in renal fibrosis. Am J Physiol Renal Physiol. 2017;313:F1-f8.
25. Tort O, Tanco S, Rocha C, Bièche I, Seixas C, Bosc C, Andrieux A, Moutin M-J, Avilés FX, Lorenzo J, et al. The cytosolic carboxypeptidases CCP2 and CCP3 catalyze posttranslational removal of acidic amino acids. Mol Biol Cell. 2014;25:3017-3027.
26. Cantalupo A, Gargiulo A, Dautaj E, Liu C, Zhang Y, Hla T and Di Lorenzo A. S1PR1 (Sphingosine-1-Phosphate Receptor 1) Signaling Regulates Blood Flow and Pressure. Hypertension. 2017;70:426-434.
27. Calabresi PA, Radue EW, Goodin D, Jeffery D, Rammohan KW, Reder AT, Vollmer T, Agius MA, Kappos L, Stites T, et al. Safety and efficacy of fingolimod in patients with relapsing-remitting multiple sclerosis (FREEDOMS II): a double-blind, randomised, placebo-controlled, phase 3 trial. Lancet Neurol. 2014;13:545-556.
28. Cantalupo A, Zhang Y, Kothiya M, Galvani S, Obinata H, Bucci M, Giordano FJ, Jiang XC, Hla T and Di Lorenzo A. Nogo-B regulates endothelial sphingolipid homeostasis to control vascular function and blood pressure. Nature medicine. 2015;21:1028-1037.
29. Azuma Y, Tan SH, Cavenagh MM, Ainsztein AM, Saitoh H and Dasso M. Expression and regulation of the mammalian SUMO-1 E1 enzyme. FASEB journal : official publication of the Federation of American Societies for Experimental Biology. 2001;15:1825-1827.
30. Tatham MH, Jaffray E, Vaughan OA, Desterro JM, Botting CH, Naismith JH and Hay RT. Polymeric chains of SUMO-2 and SUMO-3 are conjugated to protein substrates by SAE1/SAE2 and Ubc9. The Journal of biological chemistry. 2001;276:35368-35374.
31. Zhang ZB, Ruan CC, Chen DR, Zhang K, Yan C and Gao PJ. Activating transcription factor 3 SUMOylation is involved in angiotensin II-induced endothelial cell inflammation and dysfunction. J Mol Cell Cardiol. 2016;92:149-157.
32. Lee A, Jeong D, Mitsuyama S, Oh JG, Liang L, Ikeda Y, Sadoshima J, Hajjar RJ and Kho C. The role of SUMO-1 in cardiac oxidative stress and hypertrophy. Antioxid Redox Signal. 2014;21:1986-2001.

33. He Z, Zhang J, Huang Z, Du Q, Li N, Zhang Q, Chen Y and Sun Z. Sumoylation of RORgammat regulates TH17 differentiation and thymocyte development. Nat Commun. 2018;9:4870.
34. Carpino N, Turner S, Mekala D, Takahashi Y, Zang H, Geiger TL, Doherty P and Ihle JN. Regulation of ZAP-70 activation and TCR signaling by two related proteins, Sts-1 and Sts-2. Immunity. 2004;20:37-46.
35. Newman TN, Liverani E, Ivanova E, Russo GL, Carpino N, Ganea D, Safadi F, Kunapuli SP and Tsygankov AY. Members of the novel UBASH3/STS/TULA family of cellular regulators suppress T-cell-driven inflammatory responses in vivo. Immunol Cell Biol. 2014;92:837-850.
36. Hacker BM, Tomlinson JE, Wayman GA, Sultana R, Chan G, Villacres E, Disteche C and Storm DR. Cloning, chromosomal mapping, and regulatory properties of the human type 9 adenylyl cyclase (ADCY9). Genomics. 1998;50:97-104.
37. Bopp T, Becker C, Klein M, Klein-Hessling S, Palmetshofer A, Serfling E, Heib V, Becker M, Kubach J, Schmitt S, et al. Cyclic adenosine monophosphate is a key component of regulatory T cell-mediated suppression. J Exp Med. 2007;204:1303-1310.
38. Huang B, Zhao J, Lei Z, Shen S, Li D, Shen GX, Zhang GM and Feng ZH. miR-142-3p restricts cAMP production in CD4+CD25- T cells and CD4+CD25+ TREG cells by targeting AC9 mRNA. EMBO Rep. 2009;10:180-185.
39. Rautureau Y, Deschambault V, Higgins ME, Rivas D, Mecteau M, Geoffroy P, Miquel G, Uy K, Sanchez R, Lavoie V, et al. ADCY9 (Adenylate Cyclase Type 9) Inactivation Protects From Atherosclerosis Only in the Absence of CETP (Cholesteryl Ester Transfer Protein). Circulation. 2018;138:1677-1692.
40. Li Y, Baldwin TA, Wang Y, Subramaniam J, Carbajal AG, Brand CS, Cunha SR and Dessauer CW. Loss of type 9 adenylyl cyclase triggers reduced phosphorylation of Hsp20 and diastolic dysfunction. Sci Rep. 2017;7:5522.
41. Sirotkin H, O'Donnell H, DasGupta R, Halford S, St Jore B, Puech A, Parimoo S, Morrow B, Skoultchi A, Weissman SM, et al. Identification of a new human catenin gene family member (ARVCF) from the region deleted in velo-cardio-facial syndrome. Genomics. 1997;41:75-83.
42. Rappe U, Schlechter T, Aschoff M, Hotz-Wagenblatt A and Hofmann I. Nuclear ARVCF protein binds splicing factors and contributes to the regulation of alternative splicing. The Journal of biological chemistry. 2014;289:12421-12434.
43. Fang X, Ji H, Kim SW, Park JI, Vaught TG, Anastasiadis PZ, Ciesiolka M and McCrea PD. Vertebrate development requires ARVCF and p120 catenins and their interplay with RhoA and Rac. The Journal of cell biology. 2004;165:87-98.
44. Marciano DK, Brakeman PR, Lee C-Z, Spivak N, Eastburn DJ, Bryant DM, Beaudoin GM, 3rd, Hofmann I, Mostov KE and Reichardt LF. p120 catenin is required for normal renal tubulogenesis and glomerulogenesis. Development. 2011;138:2099-2109.
45. Pautsch A, Stadler N, Lohle A, Rist W, Berg A, Glocker L, Nar H, Reinert D, Lenter M, Heckel A, et al. Crystal structure of glucokinase regulatory protein. Biochemistry. 2013;52:3523-3531.
46. Grimsby J, Coffey JW, Dvorozniak MT, Magram J, Li G, Matschinsky FM, Shiota C, Kaur S, Magnuson MA and Grippo JF. Characterization of glucokinase regulatory protein-deficient mice. The Journal of biological chemistry. 2000;275:7826-7831.
47. Gu Y, Mao Y, Li H, Zhao S, Yang Y, Gao H, Yu J, Zhang X, Irwin DM, Niu G, et al. Long-term renal changes in the liver-specific glucokinase knockout mouse: implications for renal disease in maturity-onset diabetes of the young 2. Transl Res. 2011;157:111-116.
